# Supplementary material for: Knowledge, attitude, and practice of the rural community about cutaneous leishmaniasis in Wolaita zone, southern Ethiopia
Source: PLoS One. 2023 Mar 28;18(3):e0283582. doi: 10.1371/journal.pone.0283582 (PMC10047512; doi:10.1371/journal.pone.0283582)
Supplement: S1 File — (DOCX) [file pone.0283582.s001.docx]

**A questionnaire guide for eco-epidemiological study on cutaneous leishmaniasis at Wolaita Zone, Omo River catchment, south Ethiopia**

**Introduction**: This questionnaire is designed to collect information from the local community and/or households regarding the eco-epidemiology of cutaneous leishmaniasis in Wolaita Zone and the surroundings. The information gathered will be solely used for scientific research purposes. All gathered data will be systematically analyzed, and the results will be communicated to governmental and non-governmental organizations/sectors for the betterment of the community under study. All tasks in this survey will be led by a PhD student and supervised by a local promoter from the Department of Biology (Wolaita Sodo University) in the framework of the VLIR-UOS TEAM Project 2019.

Date:___/ __/_____ Time:___:___

Name of interviewer:

**1. Household socio-demographic questions**

| **No.** | **Questions and filters** | **Coding category/Answer** | **Remarks** |
| --- | --- | --- | --- |
| 1.1 | Code of the respondent |  |  |
| 1.2 | Woreda |  |  |
| 1.3 | Kebeble |  |  |
| 1.4 | Village/Site |  |  |
| 1.5 | House number |  |  |
| 1.6 | Name of respondent |  |  |
| 1.7 | Age of respondent |  |  |
| 1.8 | Sex | 1.Male 2. Female |  |
| 1.9 | Family category | 1.Father/husband  2. Mother/wife  3. Children  4. Relative  5. Other_______________ |  |
| 1.10 | Occupation | 1.Farmer  2. Student (Grade____)  3. Merchant  4. Employee (GO/NGO)  5. Other_______________ |  |
| 1.11 | Highest education level attained | 1.Non formal education  2.Primary school  3.Secondary school  4.Higher education |  |
| 1.12 | Number of persons in the household? |  |  |
| 1.13 | Family income per month | __________ Birr |  |

**2. Knowledge questions related to cutaneous leishmaniasis.**

| **No.** | **Questions and filters** | **Coding category/Answer** | **Remarks** |
| --- | --- | --- | --- |
| 2.1 | Do you know zoonosis? | 1.Yes. 2.No. |  |
| 2.2 | Do you know CL? | 1.Yes. 2.No. |  |
| 2.3 | How do you differentiate CL from other skin diseases? | 1. Mostly occur on the face  2. Disfigure the skin  3. Cause lasting wound  4. Cause pain  5. I don’t know |  |
| 2.4 | How is CL acquired/ transmitted? | 1. Contact with an infected person  2. Contact with infected animals  3. Sandfly bite  4. I don’t know |  |
| 2.5 | Do you know hyraxes? | 1.Yes. 2. No. |  |
| 2.6 | Do you know sand flies (vectors of CL)? | 1.Yes. 2. No. |  |
| 2.7 | Are hyraxes present in your area? | 1.Yes. 2.No. |  |
| 2.8 | How far is the hyrax habitat from your house? (Distance estimation) | _______ minutes of walking  _________ meters (1km=1000m) |  |
| 2.9 | How far is nearest cliff/cave/bush from your house? (Distance estimation) | _______ minutes of walking  _________ meters (1km=1000m) |  |
| 2.10 | Are all nearby rock-cliffs/caves/bushes harbor hyraxes? | 1.Yes. 2.No. |  |
| 2.11 | Do you consider hyraxes as pests? | 1.Yes. 2.No. |  |
| 2.12 | How do you compare CL with another disease like malaria? |  |  |
| 2.13 | Were you bitten by an insect other than a mosquito? | 1.Yes. (_____________)  2.No. |  |
| 2.14 | How do you know you were bitten by insects? | 1. Pricking feeling  2. Itch  3. Mark on skin  4. Other_____________ |  |
| 2.15 | In which season were you most bitten by insects? | 1. Dry season  2. Rainy season |  |
| 2.16 | At which time of the day did insects often bite you? | 1. Evening  2. Night  3. Morning  4. Afternoon |  |
| 2.17 | Place where you are often bitten by insects? | 1. Inside house  2. Household compound  3. Farm yard  4. Near rocks/caves/bushes  5. Other___________________ |  |

**3. Practice questions related to cutaneous leishmaniasis.**

| 3.1 | Where do people go for treatment in your area? | 1. Traditional healer  2. Hospital/clinic  3. Other_______________ |  |
| --- | --- | --- | --- |
| 3.2 | What type of medication do CL patients receive? | 1. Herbal medicine |  |
|  |  | 2. Burning |  |
|  |  | 3. Wholly water |  |
|  |  | 4. Modern medicine |  |
| 3.3 | Is there any practice to control/prevent CL in your area? | 1.Yes. 2.No. |  |
| 3.4 | If “yes”, who initiated the practice of control/prevention? | 1. Government/health sector  2. Community members  3. Other_______________________ |  |
| 3.5 | Do you use personal protective means against mosquito/sand fly bite? | 1.Yes. 2.No. |  |
| 3.6 | Do you own mosquito bed net? | 1.Yes. 2.No. |  |
| 3.7 | Do you regularly use the bed net? | 1.Yes. 2.No. |  |
| 3.8 | Do you think mosquito bed net can protect from sand fly bite? | 1.Yes. 2.No. |  |
| 3.9 | Is there anyone in your family with CL? | 1.Yes. 2.No. |  |
| 3.10 | Number of cases in the household? | 1. <5 years____ (active___/scar____)  2. 5-15 years___ (active___/scar____)  3. >15 years___ (active___/scar____) |  |
| 3.11 | Did you plan to control/eliminate hyraxes? | 1.Yes. 2.No. |  |
| 3.12 | Do you own dog? | 1.Yes. 2.No. |  |
| 3.13 | What do you think about using dogs for hyrax control? |  |  |
| 3.14 | Do you live with domestic animals in the same house? | 1.Yes. 2.No. |  |
| 3.15 | What type of domestic/pet animals do you have? |  |  |
| 3.16 | Type of wall of the house | 1.Wood-frame with grass thatch  2.Mud with wood-frame  3.Other_____________ |  |
| 3.17 | Where do you dispose of the waste of domestic animals? | 1. Garden/surrounding  2. Toilet  3. Other (specify)________________ |  |
| 3.18 | Do you have a separate kitchen? | 1.Yes. 2.No. |  |
|  |  |  |  |

**4. Attitude questions related to cutaneous leishmaniasis.**

| 4.1 | Do you think CL is treatable? | 1.Yes. 2.No. |  |
| --- | --- | --- | --- |
| 4.2 | Do you think CL patients should receive modern medication? | 1. Yes. 2. No. |  |
| 4.3 | Do you think CL is a health problem in your area? | 1. Yes. 2. No. |  |
| 4.4 | Do think CL is a vector-born disease? | 1.Yes. 2.No. |  |
| 4.5 | Do you feel bad when meeting CL patients? | 1.Yes. 2.No. |  |
| 4.6 | How do you feel when you meet persons with CL? | 1. Avoid contact fearing transmission  2. Don’t feel bad  3. Other_______________________ |  |
| 4.7 | Do you think CL is a stigmatizing disease? | 1.Yes. 2.No. |  |
| 4.8 | Where/how are CL patients discriminated? (Multiple response possible) | 1. Within family  2. Schooling  3. Social gatherings  4. Marriage  5. Other__________________ |  |

For each of the question below, tick (✓)the response that best characterizes how you feel about CL.

|  |  | Strongly disagree | Disagree | Undecided | Agree | Strongly agree |
| --- | --- | --- | --- | --- | --- | --- |
| 1 | CL is treatable |  |  |  |  |  |
| 2 | CL patients should receive modern medication |  |  |  |  |  |
| 3 | CL is a health problem in your area |  |  |  |  |  |
| 4 | CL is a vector-born disease |  |  |  |  |  |
| 5 | Feel bad when meeting CL patients |  |  |  |  |  |
| 6 | CL is a stigmatizing disease |  |  |  |  |  |
