# Supplementary material for: Knowledge, attitude, and practice of the rural community about cutaneous leishmaniasis in Wolaita zone, southern Ethiopia
Source: PLoS One. 2023 Mar 28;18(3):e0283582. doi: 10.1371/journal.pone.0283582 (PMC10047512; doi:10.1371/journal.pone.0283582)
Supplement: Attachment — Submitted filename: PONE-D-22-15781_reviewer_comments.pdf [file pone.0283582.s003.pdf]

PLOS ONE

Knowledge, attitude, and practice of the rural community about cutaneous leishmaniasis in Wolaita zone, southern Ethiopia

--Manuscript Draft--

**Manuscript Number:**	PONE-D-22-15781
**Article Type:**	Research Article
**Full Title:**	Knowledge, attitude, and practice of the rural community about cutaneous leishmaniasis in Wolaita zone, southern Ethiopia
**Short Title:**	Knowledge, attitude, and practice of the rural community about cutaneous leishmaniasis in Wolaita zone
**Corresponding Author:**	Bereket Alemayehu, MSc/Associate Professor<br>Wolaita Sodo University<br>Wolaita Sodo, SNNPR,Ethiopia ETHIOPIA
**Keywords:**	Cutaneous Leishmaniasis; Leishmania; knowledge; attitude; practice; Wolaita zone; Ethiopia
**Abstract:**	Background: Cutaneous leishmaniasis (CL) is a neglected tropical disease that is caused by a Leishmania parasite and transmitted by the bite of infected female sandflies. The community's understanding of CL is an essential component of disease control and prevention. This study, therefore, aimed to assess the knowledge, attitude, and practice (KAP) of the community toward CL in Wolaita zone, southern Ethiopia.<br>Methods: A community-based cross-sectional study design was employed to include 422 study subjects selected using a systematic random sampling technique from two districts, Kindo Didaye and Sodo Zuria. A pretested semi-structured questionnaire was used to collect data from the household heads. Bivariate and multivariate logistic regression analyses were performed to determine the relationship between the participants' knowledge about CL and socio-demographic characteristics.<br>Results: Out of the 422 study participants, only 19% had good knowledge on CL in general. Most, 283 (67.1%) of the respondents knew CL by its local name ("bolbo" or "moora") though this knowledge was highly varied over the study districts. The majority (86.3%) of respondents didn't know how CL is acquired, though they considered CL a health problem. Most (62.8%) respondents also believed CL was an untreatable disease. The vast majority (84.8%) of the participants had poor practice in CL prevention and treatment. Most (77%) participants responded that CL patients preferred to go to traditional healers for treatment. Herbal treatment was the most (50.2%) used, followed by burning (25.1%) to treat CL lesions.<br>Conclusion: The overall knowledge, attitude, and practice about CL and its prevention in the study area were low. This emphasizes the need to implement health education and awareness campaign to reduce the risk of CL infection. Policymakers and stakeholders should also give due attention to the prevention and treatment of CL in the study area. Further studies focusing on the eco-epidemiology of CL should be done to provide additional information to plan for CL control programs in the study area.
**Order of Authors:**	Bereket Alemayehu, MSc/Associate Professor<br>Abraham Getachew Kelbore, MSc/Assistant Professor<br>Mihiretu Alemayehu, MSc/Assistant Professor<br>Chimdesa Adugna, MSc/Lecturer<br>Tessema Bibo, BSc/MSc Fellow<br>Aberham Megaze, PhD<br>Herwig Leirs, Prof./Dr.
**Additional Information:**	
**Question**	**Response**
**Financial Disclosure**	This work was funded by the Flemish Interuniversity Council (VLIR-UOS,

Powered by Editorial Manager® and ProduXion Manager® from Aries Systems Corporation

Enter a financial disclosure statement that describes the sources of funding for the work included in this submission. Review the submission guidelines for detailed requirements. View published research articles from *PLOS ONE* for specific examples.<br><br>This statement is required for submission and **will appear in the published article** if the submission is accepted. Please make sure it is accurate.<br><br>**Unfunded studies**<br>Enter: *The author(s) received no specific funding for this work.*<br><br>**Funded studies**<br>Enter a statement with the following details:<br>• Initials of the authors who received each award<br>• Grant numbers awarded to each author<br>• The full name of each funder<br>• URL of each funder website<br>• Did the sponsors or funders play any role in the study design, data collection and analysis, decision to publish, or preparation of the manuscript?<br>• **NO** - Include this sentence at the end of your statement: *The funders had no role in study design, data collection and analysis, decision to publish, or preparation of the manuscript.*<br>• **YES** - Specify the role(s) played.<br><br>* typeset	ET2019TEA485A102). The funders had no role in study design, data collection, and analysis, decision to publish, or preparation of the manuscript.
**Competing Interests**<br><br>Use the instructions below to enter a competing interest statement for this submission. On behalf of all authors, disclose any competing interests that could be perceived to bias this work—acknowledging all financial support and any other relevant financial or non-financial competing interests.<br><br>This statement is required for submission and **will appear in the published article** if	The authors have declared that no competing interests exist.

the submission is accepted. Please make sure it is accurate and that any funding sources listed in your Funding Information later in the submission form are also declared in your Financial Disclosure statement.<br><br>View published research articles from *PLOS ONE* for specific examples.<br><br>**NO authors have competing interests**<br><br>Enter: *The authors have declared that no competing interests exist.*<br><br>**Authors with competing interests**<br><br>Enter competing interest details beginning with this statement:<br><br>*I have read the journal's policy and the authors of this manuscript have the following competing interests: [insert competing interests here]*<br><br>* typeset	
**Ethics Statement**<br><br>Enter an ethics statement for this submission. This statement is required if the study involved:<br><br>• Human participants<br>• Human specimens or tissue<br>• Vertebrate animals or cephalopods<br>• Vertebrate embryos or tissues<br>• Field research<br><br>Write "N/A" if the submission does not require an ethics statement.<br><br>General guidance is provided below. Consult the submission guidelines for detailed instructions. **Make sure that all information entered here is included in the Methods section of the manuscript.**	The study was reviewed and approved by the Institutional Review Board of Wolaita Sodo University under the reference number WSU41/12/1225. Subsequently, Permissions were obtained from Wolaita Zone Health Bureau and the respective District authorities. Written informed consent was obtained from each study participant. All demographic data of the study participants were kept confidential and anonymized before analysis.

Powered by Editorial Manager® and ProduXion Manager® from Aries Systems Corporation

**Format for specific study types**<br><br>**Human Subject Research (involving human participants and/or tissue)**<br>• Give the name of the institutional review board or ethics committee that approved the study<br>• Include the approval number and/or a statement indicating approval of this research<br>• Indicate the form of consent obtained (written/oral) or the reason that consent was not obtained (e.g. the data were analyzed anonymously)<br><br>**Animal Research (involving vertebrate animals, embryos or tissues)**<br>• Provide the name of the Institutional Animal Care and Use Committee (IACUC) or other relevant ethics board that reviewed the study protocol, and indicate whether they approved this research or granted a formal waiver of ethical approval<br>• Include an approval number if one was obtained<br>• If the study involved *non-human primates*, add *additional details* about animal welfare and steps taken to ameliorate suffering<br>• If anesthesia, euthanasia, or any kind of animal sacrifice is part of the study, include briefly which substances and/or methods were applied<br><br>**Field Research**<br><br>Include the following details if this study involves the collection of plant, animal, or other materials from a natural setting:<br>• Field permit number<br>• Name of the institution or relevant body that granted permission	
**Data Availability**<br><br>Authors are required to make all data underlying the findings described fully available, without restriction, and from the time of publication. PLOS allows rare exceptions to address legal and ethical concerns. See the PLOS Data Policy and FAQ for detailed information.	Yes - all data are fully available without restriction

A Data Availability Statement describing where the data can be found is required at submission. Your answers to this question constitute the Data Availability Statement and **will be published in the article**, if accepted.<br><br>**Important:** Stating 'data available on request from the author' is not sufficient. If your data are only available upon request, select 'No' for the first question and explain your exceptional situation in the text box.<br><br>Do the authors confirm that all data underlying the findings described in their manuscript are fully available without restriction?	
**Describe where the data may be found in full sentences. If you are copying our sample text, replace any instances of XXX with the appropriate details.**<br><br>• If the data are **held or will be held in a public repository**, include URLs, accession numbers or DOIs. If this information will only be available after acceptance, indicate this by ticking the box below. For example: *All XXX files are available from the XXX database (accession number(s) XXX, XXX.).*<br>• If the data are all contained **within the manuscript and/or Supporting Information files**, enter the following: *All relevant data are within the manuscript and its Supporting Information files.*<br>• If neither of these applies but you are able to provide **details of access elsewhere**, with or without limitations, please do so. For example:<br><br>*Data cannot be shared publicly because of [XXX]. Data are available from the XXX Institutional Data Access / Ethics Committee (contact via XXX) for researchers who meet the criteria for access to confidential data.*<br><br>*The data underlying the results presented in the study are available from (include the name of the third party*	All relevant data are within the manuscript and its Supporting Information files

and contact information or URL).<br>• This text is appropriate if the data are owned by a third party and authors do not have permission to share the data.<br><br>* typeset	
Additional data availability information:	Tick here if the URLs/accession numbers/DOIs will be available only after acceptance of the manuscript for publication so that we can ensure their inclusion before publication.

Powered by Editorial Manager® and ProduXion Manager® from Aries Systems Corporation

Manuscript

Click here to access/download;Manuscript;Manuscript_ KAP CL_Wolaita.docx

Knowledge, attitude, and practice of the rural community about cutaneous leishmaniasis in Wolaita zone, southern Ethiopia

Bereket Alemayehu[1,4*], Abraham Getachew Kelbore[2], Mihiretu Alemayehu[3], Chimdesa Adugna[1], Tessema Bibo[1], Aberham Megaze[1], Herwig Leirs[4]

[1] Department of Biology, College of Natural and Computational Sciences, Wolaita Sodo University, Wolaita Sodo, Ethiopia

[2] Department of Dermatology, College of Health Sciences and Medicine, Wolaita Sodo University, Wolaita Sodo, Ethiopia

[3] School of Public Health, College of Health Sciences and Medicine, Wolaita Sodo University, Wolaita Sodo, Ethiopia

[4] Evolutionary Ecology Group, University of Antwerp, Antwerp, Belgium

Bereket A: bereketalemayehu@gmail.com (Corresponding author)

Abraham G: kelbore2005@gmail.com

Mihiretu A: mihiretua@gmail.com

Chimdesa A: chimdesaadugna@gmail.com

Tessema B: tessemabibo2015@gmail.com

Aberham M: Abme749@yahoo.com

H Leirs: herwig.leirs@uantwerpen.be

Abstract

Background: Cutaneous leishmaniasis (CL) is a neglected tropical disease that is caused by a *Leishmania* parasite and transmitted by the bite of infected female sandflies. The community's understanding of CL is an essential component of disease control and prevention. This study, therefore, aimed to assess the knowledge, attitude, and practice (KAP) of the community toward CL in Wolaita zone, southern Ethiopia.

Methods: A community-based cross-sectional study design was employed to include 422 study subjects selected using a systematic random sampling technique from two districts, Kindo Didaye and Sodo Zuria. A pretested semi-structured questionnaire was used to collect data from the household heads. Bivariate and multivaria[illegible]ogistic regression analyses were performed to determine the relationship between the participants' knowledge about CL and socio-demographic characteristics.

Resul[illegible]ut of the 422 study participants, only 19% had good knowledge on CL in general. Most, 283 (67.1%) of the respondents knew CL by its local name ("bolbo" or "moora") though [illegible]s knowledge was highly varied over the study districts. The majority (86.3%) of respondents didn't know how CL is acquired, though they considered CL a health problem. Most (62.8%) respondents also believed CL was an untreatable disease. The vast majority (84.8%) of the participants had poor practice in CL prevention and treatment. Most (77%) participants responded that CL patients preferred to go to traditional healers for treatment. Herbal treatment was the most (50.2%) used, followed by burning (25.1%) to treat CL lesions.

Conclusion: The overall knowledge, attitude, and practice about CL and its prevention in the study area were low. This emphasizes the need to implement health education and awareness campaign to reduce the risk of CL infection. Policymakers and stakeholders should also give due

attention to the prevention and treatment of CL in the study area. Further studies focusing on the eco-epidemiology of CL should be done to provide additional information to plan for CL control programs in the study area.

Keywords: Cutaneous leishmaniasis, *Leishmania*, Knowledge, Practice, Wolaita zone, Ethiopia

Introduction

Cutaneous leishmaniasis is a neglected tropical disease and is caused by a protozoan parasite of the genus *Leishmania*. It is a vector-borne disease transmitted by the bite of infected female sandflies of various species [1]. The disease is the most common form of leishmaniasis, which causes skin lesions on the exposed parts of the body, leaving life-long scars and serious disability or stigma. WHO estimated that between 600,000 to 1 million new cases of cutaneous leishmaniasis occur worldwide annually, though this might be an underestimation due to misdiagnosis and lack of reporting. ~~About 95% of cutaneous leishmaniasis cases occurred in the Americas, the Mediterranean Basin, the Middle East, Central Asia, and the Sub-Saharan region. In 2018, of the 200 countries and territories reported to WHO to have leishmaniasis, 44% were considered endemic for cutaneous leishmaniasis [2]. In 2020, about 85% of new cases occurred in 10 countries: Afghanistan, Algeria, Brazil, Colombia, Iraq, Libya, Pakistan, Peru, the Syrian Arab Republic, and Tunisia. East African countries, including Ethiopia, are highly endemic to the cutaneous form of leishmaniasis [3].~~

~~In endemic countries, the transmission cycle of cutaneous leishmaniasis is very complex, involving various species of parasites, sandfly vectors, and reservoir hosts [4, 5]. Though over 21 species of the *Leishmania* cause human leishmaniasis, cutaneous leishmaniasis is commonly~~

caused by *L. mexicana* and *L. (Viannia) braziliensis* in the Americas, and *L. major* or *L. tropica* in all other countries, while *L. aethiopica* is the principal causative agent of cutaneous leishmaniasis in Ethiopia and a few areas in Kenya [6]. Various sandfly vectors transmit cutaneous leishmania parasites. In Africa and other Old World regions, the *Phlebotomine* sandflies are responsible for transmitting both anthroponotic and zoonotic cutaneous leishmania parasites [7, 8]. In areas where zoonotic cutaneous leishmaniasis occurs, wild and synanthropic mammalian reservoir hosts play a role in the disease transmission cycle [9, 10].

Ethiopia is one of the world's cutaneous leishmaniasis endemic countries, with an estimated incidence of 20,000 to 50,000 cases annually [11, 12]. Almost all cutaneous leishmaniasis infection in Ethiopia is caused by *L. aethiopica*. The infection due to this parasite can result from a mild, localized cutaneous leishmaniasis, to a more severe form, diffused cutaneous leishmaniasis [6, 13, 14]. The Ethiopian cutaneous leishmaniasis is reported to be a zoonotic involving either the rock hyraxes (*Procavia capensis*) or the bush hyraxes (*Heterohyrax brucei*) as reservoir hosts, and the transmission to the human host is effected through blood-feeding sandflies of the species, *P. longipes* and *P. pedifer* [4, 6, 9, 14]. Studies have reported the magnitude of cutaneous leishmaniasis and its ecology in Ethiopia until recently [4, 7, 15-17]; however, the disease has received less attention. Hence, it continued to be a public health problem in the country. Added to this, the studies related to cutaneous leishmania infection are patchy and not complete regarding eco-epidemiological coverage. Lack of knowledge about this infection among high-risk community groups also exists in the country, which may have a negative impact on the control and prevention measures [18, 19].

Understanding the disease-related knowledge, attitude, and practice (KAP) in a population is worth pushing the control and prevention activities forward in disease-endemic areas [20]. KAP studies help the health sector to design control programs in such areas. There was no previous report on the KAP about cutaneous leishmaniasis in the present study area. Therefore, the present study aimed to assess the community's knowledge, attitude, and practice about cutaneous leishmaniasis in Wolaita zone, southern Ethiopia.

Materials and methods

Study area

The study was conducted in Wolaita zone, southern Ethiopia (Fig 1). The zone is situated at a distance of 330 Km away from Addis Ababa, the Ethiopian capital. The administrative areas in Wolaita zone are structured in *woredas* (districts) and *kebeles* (villages). The village is further structured into various levels of organizations such as the Health Development Team and 'Farmers' Associations for effective implementation and monitory of the community-based agricultural and health extension programs. Wolaita zone is one of Ethiopia's most densely populated zones, with over 342 persons living per square kilometer [21]. Because of this, people inhabit all types of agroecologies, including mountainous areas. The majority of the population in the zone resides in rural districts where the primary livelihood is agriculture [22].

Figure 1. **A location map of the study area (created with ESRI ArcGIS Desktop 10.8)**.

Study design

A community-bas[illegible]oss-sectional study was conducted from August to October 2020 in two purposively selected districts, Kindo Didaye and Sodo Zuria, of Wolaita zone. The purposive selection of the districts was made after the reconnaissance survey had been conducted in the mid-highland areas of the zone. The survey aimed to have a preliminary assessment of the presence of cutaneous leishmaniasis by asking the farmer community in the rural setting and obtaining reports from health facilities. By using the Garmin 72H GPS apparatus, villages having areas above an altitude of 1700 m.a.s.l. were taken: six villages from Kindo Didaye district (Bosa Borto, Bosa Manara, Sime Dolaye, Zebo, Bereda, and Mogisa) and three villages from Sodo Zuria district (Gurumo Woide, Damot Waja, and DalboWogene) were included in this study. The purposive focus on the study areas with the specified altitude was because cutaneous leishmaniasis is reported from highland or mid-highland areas in Ethiopia [4, 9, 14, 23].

Sample size and sampling

The sample size was calculated using the single population proportion statistical formula for health studies [24], $n=Z^2 P(1-P)/d^2$,with the following assumption: n = the number of study subjects (household heads), **Z** is a critical value (1.96) at 95% confidence level, P= an anticipated population proportion assumed to be 50% to obtain a maximum sample size since there was no previously established report on the KAP about CL for the study area, d= the level of precision or margin of error (5%). Therefore, the minimum sample size of the household heads required for this study was 384. After adding a 10% non-response rate, the total sample size was calculated to be 422. The study households were selected by using a systematic sampling technique from the list of households registered at the administration offices of the corresponding villages. The household heads [illegible] then approached to gather information about

the knowledge, attitude, and practice (KAP) of the community towards cutaneous leishmaniasis and its prevention.

Data collection procedure

A total of thirty-six data collectors and nine supervisors collected the data. The two-day training was given to data collectors and supervisors [illegible]ore the data collection. Pretest was done in a neighboring village (not included in the actual data collection) in 5% of the sample size. The data were collected using a pretested structured questionnaire (S1) along with direct observation of the housing and the environmental conditions. The questionnaire contained four parts: part one related to the socio-demography of study participants, part two on knowledge regarding CL, part three on practice related to CL prevention and treatment, and part four on attitude towards CL. The questionnaire was prepared in English and then translated into Amharic or "Wolaittatto" as appropriate and back-translated into English to check for consistency.

Measurement of knowledge, attitude, and practice of participants

Participants' knowledge, attitude, and practice [illegible] measured according to the method described in other studies [18, 25, 26] with some modifications to adapt to the present disease. Each correct response to a question was assigned a score of 1, and each incorrect or unsure response was assigned a score of 0. The knowledge was assessed using a composite variable created from 5-item questions that assessed the knowledge of participants towards CL. The total knowledge scores ranged from 0 to 5. The knowledge scores between 0 and 2 were considered to indicate poor knowledge, while scores between 3 and 5 were considered to indicate good knowledge. The attitude of participants towards CL was assessed by a 5-point Likert scale that ranged from

"strongly disagree" to "strongly agree." The composite variable for attitude was created by computing the responses to 5-item questions. The total attitude scores ranged from 0 to 5. The study participants who scored between 0 and 2 were considered to have a "unfavorable attitude," while those who scored between 3 and 5 we[illegible]onsidered to have a "favorable attitude." The practice toward CL prevention and treatment was also assessed by 5-item questions. The score for practice ranged from 0 to 5, with a score of 0 to 2 indicating "poor practice," and a score of 3 to 5 indicating "good practice."

Data analysis

Data analysis was performed with R version 4.1 after entering, cleaning, and organizing the data in an Epidata version 4.6.0.2. Descriptive statistics (frequency, percentage and mean) were used to describe the socio-demographic variabl[illegible]Categorical variables were presented using frequencies and percentages. Bivariate and multivariate logistic regression analyses were performed to determine the relationship between the participants' knowledge about CL and socio-demographic characteristics. Possible associations were measured using an adjusted odds ratio (AOR) with 95% CI, and a p-value of less than 0.05 was considered statistically significant.

Ethics statement

The study was reviewed and approved by the Institutional Review Board of Wolaita Sodo University under the reference number WSU41/12/1225. Subsequently, Permissions were obtained from Wolaita Zone Health Bureau and the respective District authorities. Written informed consent was obtained from each study participant. All demographic data of the study participants were kept confidential and anonymized before analysis.

Results

Socio-demographic profiles of the participants

Of the 422 household heads who participated in this study, 367 (87%) were males, and 55 (13%) were females. The average age of respondents was 43.52 years, with a standard deviation of 10.38 years. The majority, 226 (53.6%), of the participants were aged less than or equal to 40 years. Most, 368 (87.2%), of the participants were farmers, while the rest were non-farmers. With regard to education, 288 (68.2%) of the participants hadn't had a formal education. The mean family size of the households was 5.51 (SD1.761, range 1-10), with ≤5 individuals living per household in 221 (52.4%) houses (Table 1).

Table 1. Socio-demographic profiles of the household heads.

Variables	Frequency	Percent
**Sex**		
Male	367	87.0
Female	55	13.0
**Age group (year)**		
≤40	226	53.6
>40	196	46.4
**Occupation**		
Farmer	368	87.2
Non-farmers	54	12.8
**Level of education**		
No formal e[illegible]ation	288	68.2

Formal education	134	31.8
**Family size**		
≤5	221	52.4
>5	201	47.6

Knowledge of the participants about CL

CL in the area has local names, "Bolbo" and "Moora," both meaning "disfigure." Mo[illegible]83 (67.1%) respondents knew CL by its local name though this knowledge was highly varied over the study districts. The majority, 369 (87.4%), of respondents hadn't known zoonosis in general. Though 58 (13.7%) of study participants responded to the biting of insects as a mode of CL transmission, none of them knew sandflies. Concerning signs and symptoms of CL, the participants responded that CL disfigures the skin (24.6%), causes lasting wounds (19.7%), mainly occurs on the face (17.5%), and causes pain (11.4%). Overall, of the 422 participants, only 80 (19%) had good knowledge about CL (Table 2).

[illegible]ble 2. Knowledge of the participants about cutaneous leishmaniasis in Wolaita zone, south Ethiopia.

**Variables**	**Frequency**	**%**
**Know CL**		
Yes	283	67.1
No	139	32.9
**Know zoonosis**		
Yes	53	12.6

No	369	87.4
**Know CL mode of transmission**		
Contact with an infected person	140	33.2
Contact with infected animals	5	1.2
By insect bite	58	13.7
I don't know	219	51.9
**Know sandf[illegible]**		
Yes	0	0
No	422	100
**Know the signs and symptoms of CL**		
Mostly occur on the face	74	17.5
Disfigure the skin	104	24.6
Cause lasting wound	83	19.7
Pain	48	11.4
I don't know	113	26.8
~~**Knowledge (overall)**~~		
~~Good~~	~~80~~	~~19~~
~~Poor~~	~~342~~	~~81~~

The attitude of the participants toward CL

One hundred fifty-seven (37.2%) of the participants had a positive attitude towards the treatability of CL. However, 318 (75.4%) of the participants had a negative attitude about the use

of modern medication to treat CL. The majority, 302 (71.6%), thought CL was a health problem in the area. Although 237 (56.2%) of the respondents had no bad feeling about meeting CL patients, a significant number, 185 (43.8%), of respondents had a negative attitude about meeting CL patients. One hundred twenty-three (29.1%) of the study participants thought CL was a stigmatizing disease. The overall 59.5% favorable attitude of respondent's about CL was mainly because most perceived CL as a health [illegible] not a stigmatizing disease (Table 3).

Table 3. The attitude of the respondents about CL in Wolaita zone, south Ethiopia.

**Variables**	**Frequency**	**%**
**CL is treatable**		
Yes	157	37.2
No	265	62.8
**CL patients should receive modern medication**		
Yes	104	24.6
No	318	75.4
**CL is a health problem in the area**		
Yes	302	71.6
No	120	28.4
**CL is not a stigmatizing disease**		
Yes	299	70.9
No	123	29.1
**I don't feel bad [illegible] meeting CL patients**		
Yes	237	56.2
No	185	43.8

~~**Attitude (overall)**~~		
~~Favorable~~	~~251~~	~~59.5~~
~~Unfavorable~~	~~171~~	~~40.5~~

Practice toward CL prevention and treatment

Three hundred twenty-five (77%) of the study participants responded that CL patients preferred to go to the traditional healers seeking treatment. Herbal treatment was the most used 212 (50.2%), followed by burning, 100 (25.1%), to treat CL. Four hundred three (95.5%) of the respondents lived with domestic animals in the same ho[illegible] Only 23 (5.5%) respondents owned at least a bed net. The indoor residual spray was also practiced by only 14 (3.3%) of the respondents (Table 4). Overall, the majority, 358 (84.8%) of the respondents, had poor practice toward CL treatment and prevention (Table 5).

Table 4. Practice toward CL prevention and treatment in Wolaita zone, south Ethiopia.

**Variables**	**Frequency**	**%**
**Treatment preference**		
Traditional healers	325	77
Hospitals/clinics	97	23
**Type of medication ~~do~~ CL patients receive**		
Herbal medicine	212	50.2
Burning	106	25.1
Modern medicine	104	24.6

**Living with domestic animals in the same house**		
Yes	403	95.5
No	19	4.5
**Own bed net**		
Yes	23	5.5
No	399	94.5
**Use insecticides indoor**		
Yes	14	3.3
No	408	96.7
**Practice (overall)**		
Good	64	15.2
Poor	358	84.8

Table 5. **Scores of knowledge, attitude, and practice toward CL and its prevention among the participants (n = 422)**.

**Composite variables (Total possible score)**	**KAP scores (Interpretation)**	**Frequency**	**%**
**Knowledge towards CL (6)**	0-2 (poor)	342	81
	3-5 (good)	80	19
**Attitude about CL (6)**	0-2 (unfavorable)	172	40.5
	3-5 (favorable)	251	59.5

**Practice towards CL (6)**	0-2 (poor)	358	84.8
	3-5 (good)	64	15.2

Factors associated with knowledge about CL

Both bivariate and multivariate logistic regression analyses showed that age, sex, and study districts were significantly associated with the overall knowledge of the respondents about CL (P<0.05). However, family size, occupation, and educational status were not associated with the respondents' overall knowledge. There were increased odds of having good knowledge among participants who were males (AOR=4.162, 95% CI= 1.386, 12.499), aged 40 years or below (AOR= 2.020, 95% CI= 1.121, 3.639), and those who lived in Kindo Didaye district (AOR=12.379, 95% CI= 4.737, 32.346) than their counterparts (Table 6).

Table 6. Association of knowledge about CL with the socio-demographic profiles of the respondents in Wolaita zone, south Ethiopia.

Variables	Knowledge about CL		COR (95% CI)	AOR (95% CI)	P value for AOR
	Good n (%)	Poor n (%)			
**Sex**					
Male	76 (20.7)	291 (79.3)	3.330 (1.167, 9.502)*	4.162 (1.386, 12.499)	0.011
Female	4 (7.3)	51 (92.7)	1	1	
**Age group (year)**					

≤40	58 (25.7)	168 (74.3)	2.731 (1.600, 4.660)*	2.020 (1.121, 3.639)	0.019
>40	22 (11.2)	174 (88.8)	1	1	
**Family size**					
≤5	35 (15.8)	186 (84.2)	0.652 (0.400, 1.065)	0.658 (0.385, 1.123)	0.125
>5	45 (22.4)	156 (77.6)	1	1	
**Occupation**					
Non farmer	15 (27.8)	39 (72.2)	1.793 (0.933, 3.445)	1.233 (0.549, 2.769)	0.612
Farmer	65 (17.7)	303 (82.3)	1	1	
**Education**					
Formal	32 (23.9)	102 (76.1)	1.569 (0.948, 2.596)	1.728 (0.928, 3.217)	0.085
Non formal	48 (16.7)	240 (83.3)	1	1	
**Study district**					
KindoDidaye	75 (27.7)	196 (72.3)	11.173 (4.407, 8.327)*	12.379 (4.737, 32.346)	0.000
SodoZuria	5 (3.3)	146 (96.7)	1	1	

Variables adjusted: Sex, Age group, Family size, Occupation, Education, and Village

*Significant association ($P \leq 0.05$) for COR

COR crude odds ratio, AOR adjusted odds ratio, CI confidence interval

Discussion

The present study assessed the knowledge, attitude, and practice (KAP) of the rural community about cutaneous leishmaniasis (CL) and the associated factors in Kindo Didaye and Sodo Zuria districts of Wolaita zone, southern Ethiopia. This study is perhaps the first to report the KAP on CL in the area. The present study showed that the study participants' overall knowledge about and practices toward disease prevention was poor. Nevertheless, more than half of the study participants had a favorable attitude about CL in that they perceived CL as a serious health problem but didn't consider it a stigmatizing disease.

The present study participants knew CL by its local name, either "bolbo" or "moora," meaning disfigurement of the exposed parts of the body. Almost all the community groups in Kindo Didaye district knew CL in the local name, while in Sodo Zuria district, none had the knowledge. The reasons for such differenc[illegible] usage of CL local name in the present area could be due to differences in the CL burden, presence or absence of clinical presentations, focal nature of disease prevalence, and lack of disease-related communication among the community groups. Studies conducted in CL endemic areas also reported that awareness about CL is a direct consequence of its burden [25, 27].

A very low proportion of the present study participants knew the mode of CL transmission. However, by using the local (traditional) knowledge, most participants could recognize CL based on its signs/symptoms. This finding agrees with a study done in northwest Ethiopia, where most respondents had traditional knowledge that [illegible]ed them recognize CL based on its clinical manifestations [28]. In a study done in Ochollo, a CL endemic focus in south Ethiopia, it was

reported that the rural community members knew lesions on the face as a common symptom of CL [20, 25]. A considerable proportion (34.4%) of the present participants misunderstood CL transmission as they responded that either "contact with infected people" or "contact with infected animals" was the means of CL transmission. The poor overall knowledge about CL among the present study participants might be due to their low level of education (68.2% didn't attain formal education), absence of active surveillance and inadequate case detection, absence of CL targeted health education, and inadequate disease prevention and control mechanisms in the area. The current finding is consistent with the results of most community-based KAP studies, which reported a better reflection of knowledge on CL [illegible]ptoms but poor knowledge about its transmission [20, 25, 27-29]. Similar to the finding of a cross-sectional study conducted in a district in Tigray, northern Ethiopia [27], where all respondents had never heard of sandflies, not one participant knew sandflies and their role in CL transmiss[illegible]n the present study. Contrary to our findings, studies in the Ochollo and the A[illegible]ara region reported a higher proportion, 49% and 58.1%, of knowledgeable participants about sandflies as CL vectors [20, 28]. Discrepant findings were also reported in other studies regarding the community knowledge about CL transmission [25, 30-32]. Such variations in knowledge about CL and its transmission in different countries might be due to differe[illegible] in the level of implementation of national strategic plans concerning neglected tropical diseases, including CL. Whereas, the differences in knowledge about CL among the community groups of a country could arise because of differences in the health education programs, socio-economy, and eco-epidemiological factors [26]. The community in CL endemic areas tends to better understand the disease and its transmission [27].

About three-quarters of the respondents of this study considered CL a health problem in the area. The current finding is consistent with the findings reported from northwest Ethiopia, where most participants believed that CL was a serious public health problem [28]. ~~However, in other communities, like the endemic communities of Paraguay, where about 10% of the respondents perceived the other way~~, CL may not be perceived as a serious problem [32]. The difference in perception about the impact of CL infection [illegible]t be due to differences in the burden of the disease in different areas.

Studies found that CL leads to severe psychosocial sufferings, including stigma [33, 34]. CL lesions and scars occurring on the face are of a considerable social impact due to stigma [35]. In the present study, about one-third of the participants p[illegible]ved CL a stigmatizing disease. In some communities, the level of social stigma could be much higher than the present finding [36-39]. ~~The observed avoidance of meeting patients with active CL lesions was due to the wrong perception about CL transmission that might have existed among the present community groups. The low level of education reported in this study might be a reason for such a wrong perception in the present area.~~ Similar explanations were provided based on other findings that a lack of community awareness of CL could lead to increased misunderstandings and wrong beliefs about the disease [15, 30].

About 77% of participants in this study responded that CL patients preferred to go to traditional healers than to hospitals or clinics to receive treatment. This finding agrees with the reported higher practices of traditional treatment for CL elsewhere among rural communities of Ethiopia: 68.3% in the Amhara region (northwest Ethiopia) [28], 90% in a district in the Tigrai region

(north Ethiopia) [27], and 67.6% in Gamo Gofa zone (south Ethiopia) [20]. However, the extent of the use of traditional medication to treat CL could be low in some communities. ~~In Shara'b district of southwestern Yemen, a CL endemic foci, only one-third of the respondents thought that CL could be treated by herbal medicine, although the actual practice of the herbal medication was not reported among that community [25].~~ The differences in the treatment practices of CL among communities in various localities might be due to a lack of community awareness of the available treatment options, accessibility of modern CL treatment centers, the socio-economy, and socio-cultural factors. In localities like the present study area, where modern CL treatment is unavailable, CL patients remain dependent on traditional therapies. The use of herbal preparations and burning CL lesions with red hot metal or clay were the widely practiced traditional methods to treat CL in the present study area. Burning of CL lesions was widely practiced in Kndo Didaye district, where most affected community groups live. ~~A few studies in Ethiopia also reported the practice of burning CL lesions [4, 20], though herbal medicine has long been used and is common in other areas, too [4, 25, 40, 41].~~

The very low (15.2%) prevention practices found in the current study coupled with the absence of proper CL treatment implies CL is a highly neglected disease in the area. These might have also resulted in the observed 62.8% negative attitude toward CL treatability. This finding is supported by a study from Yemen [25], where the absence of treatment facilities resulted in an increased negative attitude about CL treatability. Community awareness about the treatability of CL might be high in areas with established leishmaniasis treatment centers [28, 42, 43]. However, the presence of a leishmaniasis treatment center alone may not be sufficient to bring a positive attitude towards CL treatability in endemic areas [20].

In this study, the knowledge about CL was found to be associated with sex, age, and study districts. The male participants had better knowledge as compared to their female counterparts. In a male-dominated rural community [44], males might have better exposure to knowledge about CL. ~~In an endemic area of Yemen [25], a similar finding was reported that male participants had better knowledge about CL~~. Males were also found more knowledgeable in a KAP study about visceral leishmaniasis in the west Armachiho district, Northwest Ethiopia [45]. However, the association of knowledge about CL with gender was reported differently in other studies in which females had better knowledge about the disease [27, 46]. In the current study, participants aged below or equal to 40 years had better knowledge. This finding agrees with a study done in Bihar, India, where the same age group had a better understanding of CL [18]. Interestingly, this study found that district was the most important factor associated with CL knowledge. Participants of the Kindo Didaye district had better knowledge than the other district. The observed district-wise difference in the Knowledge about CL might be attributed to the eco-epidemiological factors and the associated experiences of the local communities regarding the disease [16, 42].

Conclusion

In this study, poor overall knowledge about and practices toward CL was found. The insufficient knowledge and community practice regarding the infection nature, vector, transmission ways, and treatment and presentation of CL emphasize the need for health education and community mobilization campaign to increase community awareness about the disease in the area. The misconceptions observed about CL treatment also need the urgent establishment of treatment

facilities. ~~Eco-epidemiological studies concerning CL should be conducted in the study area to provide more information about the disease and initiate control programs.~~

List of abbreviations

CL: Cutaneous Leishmaniasis; KAP: Knowledge, Attitude, and Practice; WHO: World Health Organization; GPS: Global Positioning System

Declarations

Ethics statement

The study was reviewed and approved by the Institutional Review Board of Wolaita Sodo University under the reference number WSU41/12/1225. Subsequently, Permissions were obtained from Wolaita Zone Health Bureau and the respective District authorities. Written informed consent was obtained from each study participant. All demographic data of the study participants were kept confidential and anonymized before analysis.

Consent for publication

Consent to publish this manuscript from the participants was deemed not applicable since the manuscript does not contain identifying data from any individual person.

Availability of data and material

The datasets used and analyzed during the current study are available from the corresponding author on reasonable request.

Competing interest

All authors declared that they have no competing interests.

Funding

This work was funded by the Flemish Interuniversity Council (VLIR-UOS, ET2019TEA485A102). The funders had no role in study design, data collection, and analysis, decision to publish, or preparation of the manuscript.

Author contributions

Conceptualization: Bereket Alemayehu, Herwig Leirs.

Data curation: Bereket Alemayehu, Chimdesa Adugna, Mihiretu Alemayehu.

Formal analysis: Bereket Alemayehu, Mihiretu Alemayehu.

Funding acquisition: Herwig Leirs.

Investigation: Bereket Alemayehu, Chimdesa Adugna, Tessema Bibo.

Methodology: Bereket Alemayehu, Abraham Getachew, Mihiretu Alemayehu, Aberham Megaze, Herwig Leirs.

Project administration: Bereket Alemayehu, Aberham Megaze, Herwig Leirs.

Resources: Aberham Megaze, Herwig Leirs.

Supervision: Bereket Alemayehu, Aberham Megaze, Herwig Leirs.

Writing – original draft: Bereket Alemayehu.

Writing – review & editing: Bereket Alemayehu, Abraham Getachew, Mihiretu Alemayehu, Chimdesa Adugna, Tessema Bibo, Aberham Megaze, Herwig Leirs.

Acknowledgments

We would like to thank VLIR-UOS, the University of Antwerp, Belgium, and Wolaita Sodo Universities for financial, technical, and administrative support. We also extend our gratitude to the respective district administrative bodies, data collectors, data clerks, local community, and study participants for their cooperation and technical assistance.

References

1. WHO. Cutaneous leishmaniasis control in selected countries of the WHO eastern Mediterranean and African regions. Report of an interregional network meeting Casablanca, Morocco 23–24 June 2014.; 2014.
2. World Health Organization. Global leishmaniasis surveillance, 2017–2018, and first report on 5 additional indicators – Surveillance mondiale de la leishmaniose, 2017-2018, et premier rapport sur 5 indicateurs supplémentaires. Wkly Epidemiol Rec. 2020;95(25):265-79.
3. WHO. World Health Organization Technical Report Series 949. Control of the leishmaniasis 2010.
4. Ashford RW, Bray MA, Hutchinson MP, Bray RS. The epidemiology of cutaneous leishmaniasis in Ethiopia. Trans R Soc Trop Med Hyg. 1973;67(4):568-601 DOI: 10.1016/0035-9203(73)90088-6.
5. Alemayehu B, Alemayehu M. Leishmaniasis: A Review on Parasite, Vector and Reservoir Host. Health Science Journal. 2017;11(4) DOI: 10.21767/1791-809x.1000519.

6. Bray RS, Ashford RW, Bray MA. The parasite causing cutaneous leishmaniasis in Ethiopia. Transactions of The Royal Society of Tropical Medicine and Hygiene. 1973;67(3):345-8 DOI: 10.1016/0035-9203(73)90111-9.

7. Pareyn M, Van den Bosch E, Girma N, van Houtte N, Van Dongen S, Van der Auwera G, et al. Ecology and seasonality of sandflies and potential reservoirs of cutaneous leishmaniasis in Ochollo, a hotspot in southern Ethiopia. PLoS Negl Trop Dis. 2019;13(8):e0007667 DOI: 10.1371/journal.pntd.0007667.

8. Killick-Kendrick R, Tang Y, Killick-Kendrick M, Johnson RN, Ngumbi PM, Sang DK, et al. Phlebotomine sandflies of Kenya (Diptera: Psychodidae). III. The identification and distribution of species of the subgenus Larroussius. Ann Trop Med Parasitol. 1994;88(2):183-96 DOI: 10.1080/00034983.1994.11812857.

9. Lemma W, Erenso G, Gadisa E, Balkew M, Gebre-Michael T, Hailu A. A zoonotic focus of cutaneous leishmaniasis in Addis Ababa, Ethiopia. Parasit Vectors. 2009;2(1):60 DOI: 10.1186/1756-3305-2-60.

10. Gebremichael D. Zoonotic impact and epidemiological changes of leishmaniasis in Ethiopia. Open Vet J. 2018;8(4):432-40 DOI: 10.4314/ovj.v8i4.13.

11. Leishmaniases WHOECotCot, World Health O. Control of the leishmaniases: report of a meeting of the WHO Expert Commitee on the Control of Leishmaniases, Geneva, 22-26 March 2010. Geneva: World Health Organization; 2010.

12. Deribe K, Meribo K, Gebre T, Hailu A, Ali A, Aseffa A, et al. The burden of neglected tropical diseases in Ethiopia, and opportunities for integrated control and elimination. Parasites & Vectors. 2012;5(1):240 DOI: 10.1186/1756-3305-5-240.

13. Hailu A, Di Muccio T, Abebe T, Hunegnaw M, Kager PA, Gramiccia M. Isolation of Leishmania tropica from an Ethiopian cutaneous leishmaniasis patient. Trans R Soc Trop Med Hyg. 2006;100(1):53-8 DOI: 10.1016/j.trstmh.2005.04.017.

14. Lemma A, Foster WA, Gemetchu T, Preston PM, Bryceson A, Minter DM. Studies on leishmaniasis in Ethiopia. I. Preliminary investigations into the epidemiology of cutaneous leishmaniasis in the highlands. Ann Trop Med Parasitol. 1969;63(4):455-72

15. Eshetu B, Mamo H. Cutaneous leishmaniasis in north-central Ethiopia: trend, clinical forms, geographic distribution, and determinants. Tropical Medicine and Health. 2020;48

16. Bsrat A, Berhe N, Balkew M, Yohannes M, Teklu T, Gadisa E, et al. Epidemiological study of cutaneous leishmaniasis in Saesie Tsaeda-emba district, eastern Tigray, northern Ethiopia. Parasit Vectors. 2015;8:149 DOI: 10.1186/s13071-015-0758-9.

17. Negera E, Gadisa E, Yamuah L, Engers H, Hussein J, Kuru T, et al. Outbreak of cutaneous leishmaniasis in Silti woreda, Ethiopia: risk factor assessment and causative agent identification. Trans R Soc Trop Med Hyg. 2008;102(9):883-90 DOI: 10.1016/j.trstmh.2008.03.021.

18. J. S D, Gupta AK, Veeri RB, Garapati P, Kumar R, Dhingra S, et al. Knowledge, attitude and practices towards visceral leishmaniasis among HIV patients: A cross-sectional study from Bihar, India. PLoS One. 2021;16(8):e0256239 DOI: 10.1371/journal.pone.0256239.

19. Vázquez ML, Kroeger A, Lipowsky R, Alzate A. [Popular conceptions regarding cutaneous leishmaniasis in Colombia and their applicability in control programs]. Bol Oficina Sanit Panam. 1991;110(5):402-12

20. Kebede N, Worku A, Ali A, Animut A, Negash Y, Gebreyes WA, et al. Community knowledge, attitude and practice towards cutaneous leishmaniasis endemic area Ochello,

Gamo Gofa Zone, South Ethiopia. Asian Pacific Journal of Tropical Biomedicine. 2016;6(7):562-7 DOI: https://doi.org/10.1016/j.apjtb.2016.01.018.

21. Wolaita Zone. Annual Socio-economic and Demographic Reports. Wolaita Sodo, Process DDCaDC; 2015.

22. Cochrane L, Gecho Y. Data on the demographics, education, health and infrastructure: Wolaita Zone, Ethiopia. Data Brief. 2018;21:2095-102 DOI: 10.1016/j.dib.2018.11.063.

23. Pareyn M, Rutten A, Merdekios B, Wedegärtner REM, Girma N, Regelbrugge L, et al. High-resolution habitat suitability model for Phlebotomus pedifer, the vector of cutaneous leishmaniasis in southwestern Ethiopia. Parasites & Vectors. 2020;13(1):467 DOI: 10.1186/s13071-020-04336-3.

24. Lwanga SK, Lemeshow S, World Health O. Sample size determination in health studies : a practical manual / S. K. Lwanga and S. Lemeshow. Geneva: World Health Organization; 1991.

25. Alharazi TH, Haouas N, Al-Mekhlafi HM. Knowledge and attitude towards cutaneous leishmaniasis among rural endemic communities in Shara'b district, Taiz, southwestern Yemen. BMC Infect Dis. 2021;21(1):269 DOI: 10.1186/s12879-021-05965-4.

26. Alemu A, Alemu A, Esmael N, Dessie Y, Hamdu K, Mathewos B, et al. Knowledge, attitude and practices related to visceral leishmaniasis among residents in Addis Zemen town, South Gondar, Northwest Ethiopia. BMC Public Health. 2013;13:382 DOI: 10.1186/1471-2458-13-382.

27. Tesfay K, Mardu F, Berhe B, Negash H, Legese H, Adhanom G, et al. Household knowledge, practice and treatment seeking behaviors towards cutaneous leishmaniasis in the endemic rural communities of Ganta- afeshum district, Tigrai, northern Ethiopia, 2019:

a cross-sectional study. Tropical Diseases, Travel Medicine and Vaccines. 2021;7(1):19 DOI: 10.1186/s40794-021-00144-4.

28. Tamiru HF, Mashalla YJ, Mohammed R, Tshweneagae GT. Cutaneous leishmaniasis a neglected tropical disease: community knowledge, attitude and practices in an endemic area, Northwest Ethiopia. BMC Infectious Diseases. 2019;19(1):855 DOI: 10.1186/s12879-019-4506-1.

29. Sarkari B, Qasem A, Shafaf MR. Knowledge, attitude, and practices related to cutaneous leishmaniasis in an endemic focus of cutaneous leishmaniasis, Southern Iran. Asian Pac J Trop Biomed. 2014;4(7):566-9 DOI: 10.12980/apjtb.4.2014c744.

30. Akram A, Khan HAA, Qadir A, Sabir AM. A Cross-Sectional Survey of Knowledge, Attitude and Practices Related to Cutaneous Leishmaniasis and Sand Flies in Punjab, Pakistan. PLOS ONE. 2015;10(6):e0130929 DOI: 10.1371/journal.pone.0130929.

31. Irum S, Aftab M, Khan A, Naz S, Simsek S, Habib A, et al. Cutaneous Leishmaniasis (CL): A Cross-Sectional Community Based Survey on Knowledge, Attitude and Practices in a Highly Endemic Area of Waziristan (KPK Province), Pakistan. Acta Trop. 2021;213:105746 DOI: 10.1016/j.actatropica.2020.105746.

32. Ruoti M, Oddone R, Lampert N, Orué E, Miles MA, Alexander N, et al. Mucocutaneous Leishmaniasis: Knowledge, Attitudes, and Practices Among Paraguayan Communities, Patients, and Health Professionals. J Trop Med. 2013;2013:538629 DOI: 10.1155/2013/538629.

33. Ramdas S, van der Geest S, Schallig HD. Nuancing stigma through ethnography: the case of cutaneous leishmaniasis in Suriname. Soc Sci Med. 2016;151:139-46 DOI: 10.1016/j.socscimed.2015.12.044.

34. Gómez LJ, van Wijk R, van Selm L, Rivera A, Barbosa MC, Parisi S, et al. Stigma, participation restriction and mental distress in patients affected by leprosy, cutaneous leishmaniasis and Chagas disease: a pilot study in two co-endemic regions of eastern Colombia. Transactions of The Royal Society of Tropical Medicine and Hygiene. 2020;114(7):476-82 DOI: 10.1093/trstmh/trz132.
35. Yanik M, Gurel MS, Simsek Z, Kati M. The psychological impact of cutaneous leishmaniasis. Clin Exp Dermatol. 2004;29(5):464-7 DOI: 10.1111/j.1365-2230.2004.01605.x.
36. Bailey F, Mondragon-Shem K, Hotez P, Ruiz-Postigo JA, Al-Salem W, Acosta-Serrano A, et al. A new perspective on cutaneous leishmaniasis-Implications for global prevalence and burden of disease estimates. PLoS Negl Trop Dis. 2017;11(8):e0005739 DOI: 10.1371/journal.pntd.0005739.
37. Bennis I, De Brouwere V, Belrhiti Z, Sahibi H, Boelaert M. Psychosocial burden of localised cutaneous Leishmaniasis: a scoping review. BMC Public Health. 2018;18(1):358 DOI: 10.1186/s12889-018-5260-9.
38. Chahed MK, Bellali H, Ben Jemaa S, Bellaj T. Psychological and Psychosocial Consequences of Zoonotic Cutaneous Leishmaniasis among Women in Tunisia: Preliminary Findings from an Exploratory Study. PLoS Negl Trop Dis. 2016;10(10):e0005090 DOI: 10.1371/journal.pntd.0005090.
39. Garapati P, Pal B, Siddiqui NA, Bimal S, Das P, Murti K, et al. Knowledge, stigma, health seeking behaviour and its determinants among patients with post kalaazar dermal leishmaniasis, Bihar, India. PLoS One. 2018;13(9):e0203407 DOI: 10.1371/journal.pone.0203407.

40. Weigel MM, Armijos RX. The traditional and conventional medical treatment of cutaneous leishmaniasis in rural Ecuador. Rev Panam Salud Publica. 2001;10(6):395-404 DOI: 10.1590/s1020-49892001001200005.

41. Weigel MM, Armijos RX, Racines RJ, Zurita C, Izurieta R, Herrera E, et al. Cutaneous leishmaniasis in subtropical Ecuador: popular perceptions, knowledge, and treatment. Bull Pan Am Health Organ. 1994;28(2):142-55

42. Yohannes M. <++Prevalence and environmental determinants of cutaneous leishmaniasis in rural communities in Tigray, northern Ethiopia journal.pntd.0007722.pdf>. DOI: 10.1371/journal

10.1371/journal.pntd.0007722.

43. Padovese V, Terranova M, Toma L, Barnabas GA, Morrone A. Cutaneous and mucocutaneous leishmaniasis in Tigray, northern Ethiopia: clinical aspects and therapeutic concerns. Transactions of The Royal Society of Tropical Medicine and Hygiene. 2009;103(7):707-11 DOI: 10.1016/j.trstmh.2009.02.023.

44. Lailulo YA, Susuman AS, Blignaut R. Correlates of gender characteristics, health and empowerment of women in Ethiopia. BMC women's health. 2015;15:116- DOI: 10.1186/s12905-015-0273-3.

45. Melkamu HT, Beyene AM, Zegeye DT. Knowledge, attitude and practices of the resident community about visceral leishmaniasis in West Armachiho district, Northwest Ethiopia. Heliyon. 2020;6(1):e03152 DOI: 10.1016/j.heliyon.2019.e03152.

46. Dires A, Kumar P, Gedamu S, Yimam W, Ademe S. Knowledge, attitude and prevention measures of students towards cutaneous leishmaniasis in Delanta district, Northeast

Ethiopia. Parasite Epidemiology and Control. 2022;17:e00241 DOI: https://doi.org/10.1016/j.parepi.2022.e00241.

Figure
Click here to access/download;Figure;Figure 1_Map of the study area.docx

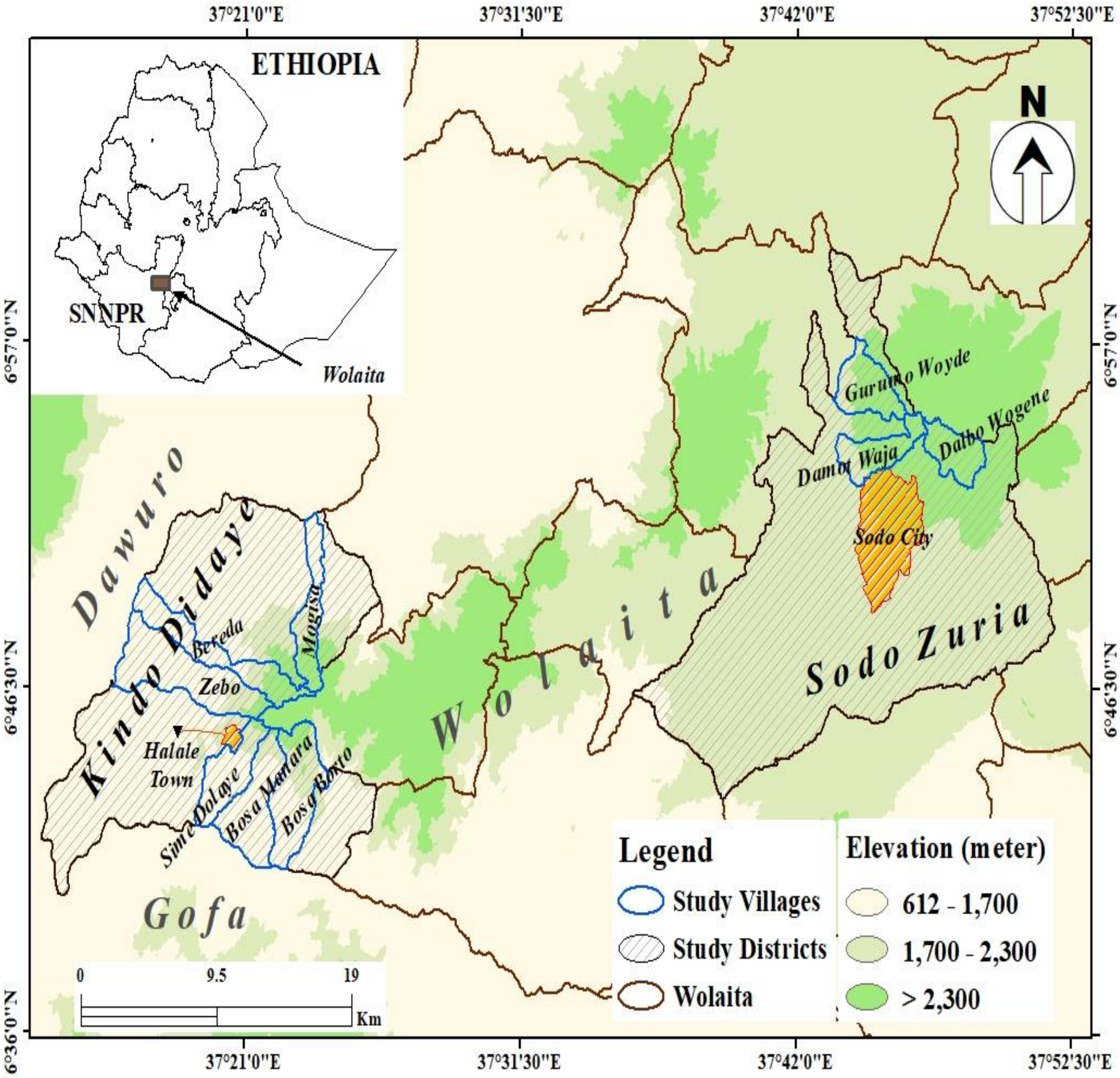

Figure 1. A location m[illegible]f the study area (created with ESRI ArcGIS Desktop 10.8).

Click here to access/download

Supporting Information

Supporting Information 1_Questionnaire.docx
