# Supplementary material for: Knowledge, attitude, and practice of the rural community about cutaneous leishmaniasis in Wolaita zone, southern Ethiopia
Source: PLoS One. 2023 Mar 28;18(3):e0283582. doi: 10.1371/journal.pone.0283582 (PMC10047512; doi:10.1371/journal.pone.0283582)
Supplement: Attachment — Submitted filename: PONE-D-22-15781_R1_comments.pdf [file pone.0283582.s005.pdf]

PLOS ONE

Knowledge, attitude, and practice of the rural community about cutaneous leishmaniasis in Wolaita zone, southern Ethiopia

--Manuscript Draft--

**Manuscript Number:**	PONE-D-22-15781R1
**Article Type:**	Research Article
**Full Title:**	Knowledge, attitude, and practice of the rural community about cutaneous leishmaniasis in Wolaita zone, southern Ethiopia
**Short Title:**	Knowledge, attitude, and practice of the rural community about cutaneous leishmaniasis in Wolaita zone
**Corresponding Author:**	Bereket Alemayehu, MSc/Associate Professor<br>Wolaita Sodo University<br>Wolaita Sodo, SNNPR,Ethiopia ETHIOPIA
**Keywords:**	Cutaneous leishmaniasis, Leishmania, Knowledge, Practice, Wolaita zone, Ethiopia
**Abstract:**	Background: Cutaneous leishmaniasis (CL) is a neglected tropical disease that is caused by a Leishmania parasite and transmitted by the bite of infected female sandflies. Community awareness is an essential component of disease control and prevention. Therefore, this study aimed to assess the community's knowledge, attitude, and practice toward CL in Wolaita zone, southern Ethiopia.<br>Methods: A community-based cross-sectional study design was employed to include 422 study subjects selected using a systematic sampling technique from two districts, Kindo Didaye and Sodo Zuria. A pretested structured questionnaire was used to collect data from the household heads. Bivariate and multivariate logistic regression analyses were performed to determine the relationship between the participants' knowledge about CL and socio-demographic characteristics.<br>Results: Out of the 422 study participants, only 19% had good knowledge of CL in general. Most (67.1%) of the respondents knew CL by its local name ("bolbo" or "moora") though this knowledge varied highly over the study districts. The majority (86.3%) of respondents did not know how CL is acquired, though they considered CL a health problem. Most (62.8%) respondents believed that CL was an untreatable disease. Most (77%) participants responded that CL patients preferred to go to traditional healers for treatment. Herbal treatment was the most (50.2%) used, followed by burning (25.1%). Knowledge about CL was significantly associated with sex, age, and study districts.<br>Conclusion: The overall knowledge, attitude, and practice about CL and its prevention in the study area were low. This emphasizes the need to implement health education and awareness campaign to reduce the risk of CL infection. Policymakers and stakeholders should also give due attention to the prevention and treatment of CL in the study area.
**Order of Authors:**	Bereket Alemayehu, MSc/Associate Professor
	Abraham Getachew Kelbore, MSc/Assistant Professor
	Mihiretu Alemayehu, MSc/Assistant Professor
	Chimdesa Adugna, MSc/Lecturer
	Tessema Bibo, BSc/MSc Fellow
	Aberham Megaze, PhD
	Herwig Leirs, Prof./Dr.
**Response to Reviewers:**	All comments of the reviewers and the editor are addressed and attached in a separate file (Response to Reviewers).
**Additional Information:**	
**Question**	**Response**
**Financial Disclosure**	This work was funded by the Flemish Interuniversity Council (VLIR-UOS,

Powered by Editorial Manager® and ProduXion Manager® from Aries Systems Corporation

Enter a financial disclosure statement that describes the sources of funding for the work included in this submission. Review the submission guidelines for detailed requirements. View published research articles from *PLOS ONE* for specific examples.<br><br>This statement is required for submission and **will appear in the published article** if the submission is accepted. Please make sure it is accurate.<br><br>**Unfunded studies**<br>Enter: *The author(s) received no specific funding for this work.*<br><br>**Funded studies**<br>Enter a statement with the following details:<br>• Initials of the authors who received each award<br>• Grant numbers awarded to each author<br>• The full name of each funder<br>• URL of each funder website<br>• Did the sponsors or funders play any role in the study design, data collection and analysis, decision to publish, or preparation of the manuscript?<br>• **NO** - Include this sentence at the end of your statement: *The funders had no role in study design, data collection and analysis, decision to publish, or preparation of the manuscript.*<br>• **YES** - Specify the role(s) played.<br><br>* typeset	ET2019TEA485A102). The funders had no role in study design, data collection, and analysis, decision to publish, or preparation of the manuscript.
**Competing Interests**<br><br>Use the instructions below to enter a competing interest statement for this submission. On behalf of all authors, disclose any competing interests that could be perceived to bias this work—acknowledging all financial support and any other relevant financial or non-financial competing interests.<br><br>This statement is required for submission and **will appear in the published article** if	The authors have declared that no competing interests exist.

the submission is accepted. Please make sure it is accurate and that any funding sources listed in your Funding Information later in the submission form are also declared in your Financial Disclosure statement.<br><br>View published research articles from *PLOS ONE* for specific examples.<br><br>**NO authors have competing interests**<br><br>Enter: *The authors have declared that no competing interests exist.*<br><br>**Authors with competing interests**<br><br>Enter competing interest details beginning with this statement:<br><br>*I have read the journal's policy and the authors of this manuscript have the following competing interests: [insert competing interests here]*<br><br>* typeset	
**Ethics Statement**<br><br>Enter an ethics statement for this submission. This statement is required if the study involved:<br><br>• Human participants<br>• Human specimens or tissue<br>• Vertebrate animals or cephalopods<br>• Vertebrate embryos or tissues<br>• Field research<br><br>Write "N/A" if the submission does not require an ethics statement.<br><br>General guidance is provided below. Consult the submission guidelines for detailed instructions. **Make sure that all information entered here is included in the Methods section of the manuscript.**	The study was reviewed and approved by the Institutional Review Board of Wolaita Sodo University under the reference number WSU41/12/1225. Subsequently, Permissions were obtained from Wolaita Zone Health Bureau and the respective District authorities. Written informed consent was obtained from each study participant. All demographic data of the study participants were kept confidential and anonymized before analysis.

**Format for specific study types**<br><br>**Human Subject Research (involving human participants and/or tissue)**<br>• Give the name of the institutional review board or ethics committee that approved the study<br>• Include the approval number and/or a statement indicating approval of this research<br>• Indicate the form of consent obtained (written/oral) or the reason that consent was not obtained (e.g. the data were analyzed anonymously)<br><br>**Animal Research (involving vertebrate animals, embryos or tissues)**<br>• Provide the name of the Institutional Animal Care and Use Committee (IACUC) or other relevant ethics board that reviewed the study protocol, and indicate whether they approved this research or granted a formal waiver of ethical approval<br>• Include an approval number if one was obtained<br>• If the study involved *non-human primates*, add *additional details* about animal welfare and steps taken to ameliorate suffering<br>• If anesthesia, euthanasia, or any kind of animal sacrifice is part of the study, include briefly which substances and/or methods were applied<br><br>**Field Research**<br><br>Include the following details if this study involves the collection of plant, animal, or other materials from a natural setting:<br>• Field permit number<br>• Name of the institution or relevant body that granted permission	
**Data Availability**<br><br>Authors are required to make all data underlying the findings described fully available, without restriction, and from the time of publication. PLOS allows rare exceptions to address legal and ethical concerns. See the PLOS Data Policy and FAQ for detailed information.	Yes - all data are fully available without restriction

A Data Availability Statement describing where the data can be found is required at submission. Your answers to this question constitute the Data Availability Statement and **will be published in the article**, if accepted.<br><br>**Important:** Stating 'data available on request from the author' is not sufficient. If your data are only available upon request, select 'No' for the first question and explain your exceptional situation in the text box.<br><br>Do the authors confirm that all data underlying the findings described in their manuscript are fully available without restriction?	
**Describe where the data may be found in full sentences. If you are copying our sample text, replace any instances of XXX with the appropriate details.**<br><br>• If the data are **held or will be held in a public repository**, include URLs, accession numbers or DOIs. If this information will only be available after acceptance, indicate this by ticking the box below. For example: *All XXX files are available from the XXX database (accession number(s) XXX, XXX.*).<br>• If the data are all contained **within the manuscript and/or Supporting Information files**, enter the following: *All relevant data are within the manuscript and its Supporting Information files.*<br>• If neither of these applies but you are able to provide **details of access elsewhere**, with or without limitations, please do so. For example:<br><br>*Data cannot be shared publicly because of [XXX]. Data are available from the XXX Institutional Data Access / Ethics Committee (contact via XXX) for researchers who meet the criteria for access to confidential data.*<br><br>*The data underlying the results presented in the study are available from (include the name of the third party*	All relevant data are within the manuscript and its Supporting Information files

and contact information or URL).<br>• This text is appropriate if the data are owned by a third party and authors do not have permission to share the data.<br><br>* typeset	
Additional data availability information:	

Powered by Editorial Manager® and ProduXion Manager® from Aries Systems Corporation

Manuscript Click here to access/download;Manuscript;Manuscript.docx

Knowledge, attitude, and practice of the rural community about cutaneous leishmaniasis in Wolaita zone, southern Ethiopia

Bereket Alemayehu[1,2*], Abraham Getachew Kelbore[3], Mihiretu Alemayehu[4], Chimdesa Adugna[1], Tessema Bibo[1], Aberham Megaze[1], Herwig Leirs[2]

[1] Department of Biology, College of Natural and Computational Sciences, Wolaita Sodo University, Wolaita Sodo, Ethiopia

[2] Evolutionary Ecology Group, University of Antwerp, Antwerp, Belgium

[3] Department of Dermatology, College of Health Sciences and Medicine, Wolaita Sodo University, Wolaita Sodo, Ethiopia

[4] School of Public Health, College of Health Sciences and Medicine, Wolaita Sodo University, Wolaita Sodo, Ethiopia

Bereket A: bereketalemayehu@gmail.com (Corresponding author)
Abraham G: kelbore2005@gmail.com
Mihiretu A: mihiretua@gmail.com
Chimdesa A: chimdesaadugna@gmail.com
Tessema B: tessemabibo2015@gmail.com
Aberham M: Abme749@yahoo.com
H Leirs: herwig.leirs@uantwerpen.be

Abstract

Background: Cutaneous leishmaniasis (CL) is a neglected tropical disease that is caused by a *Leishmania* parasite and transmitted by the bite of infected female sandflies. Community awareness is an essential component of disease control and prevention. Therefore, this study

aimed to assess the community's knowledge, attitude, and practice toward CL in Wolaita zone, southern Ethiopia.

Methods: A community-based cross-sectional study design was employed to include 422 study subjects selected using a systematic sampling technique from two districts, Kindo Didaye and Sodo Zuria. A pretested structured questionnaire was used to collect data from the household heads. Bivariate and multivariate logistic regression analyses were performed to determine the relationship between the participants' knowledge about CL and socio-demographic characteristics.

Results: Out of the 422 study participants, only 19% had good knowledge of CL in general. Most (67.1%) of the respondents knew CL by its local name ("bolbo" or "moora") though this knowledge varied highly over the study districts. The majority (86.3%) of respondents did not know how CL is acquired, though they considered CL a health problem. Most (62.8%) respondents believed that CL was an untreatable disease. Most (77%) participants responded that CL patients preferred to go to traditional healers for treatment. Herbal treatment was the most (50.2%) used, followed by burning (25.1%). Knowledge about CL was significantly associated with sex, age, and study districts.

Conclusion: The overall knowledge, attitude, and practice about CL and its prevention in the study area were low. This emphasizes the need to implement health education and awareness campaign to reduce the risk of CL infection. Policymakers and stakeholders should also give due attention to the prevention and treatment of CL in the study area.

Keywords: Cutaneous leishmaniasis, *Leishmania*, Knowledge, Practice, Wolaita zone, Ethiopia

Introduction

Cutaneous leishmaniasis (CL) is a neglected tropical disease (NTD) and is caused by a protozoan parasite of the genus *Leishmania*. It is a vector-borne disease transmitted by the bite of infected female sandflies of various species [1]. The disease is the most common form of leishmaniasis, which causes skin lesions on the exposed parts of the body, leaving life-long scars and serious disability or stigma. WHO estimated that 600,000 to 1 million new cases of CL occur worldwide annually, though this might be an underestimation due to misdiagnosis and lack of reporting. About 95% of CL cases occurred in the Americas, the Mediterranean Basin, the Middle East, Central Asia, and the Sub-Saharan region. In 2018, of the 200 countries and territories reported to WHO to have leishmaniasis, 44% were considered endemic for CL [2]. In endemic countries, the transmission cycle of CL is very complex, involving various species of parasites, sandfly vectors, and reservoir hosts [3, 4].

Ethiopia is one of the world's CL endemic countries, with an estimated incidence of 20,000 to 50,000 cases annually [5, 6]. Almost all CL infection in Ethiopia is caused by a *L. aethiopica* parasite. Infection with this parasite can result in a mild localized CL to a more severe form, diffused CL [7-9]. The Ethiopian CL is reported to be zoonotic, involving hyraxes as reservoir hosts, and the transmission to the human host is effected through blood-feeding *Phlebotomine* sandflies [3, 8-10].

Studies have reported the magnitude of CL and its ecology in Ethiopia until recently [3, 11-14]. However, the disease continues to be a public health problem in the country. There is a lack of knowledge about CL infection among communities in the country, which hinders community

participation in disease prevention and control [15, 16]. The national master plan of NTDs of Ethiopia has prioritized community engagement as one of the core elements to achieve the national goals of preventing and controlling NTDs, including CL [17].

Rural communities bear the highest disease burden [18-20]. The lack of health facilities and inaccessibility of appropriate treatments worsen the situation [21]. Due to a lack of awareness, there are also misconceptions about CL among remote communities [22]. Community-based knowledge, attitude, and practice (KAP) studies are therefore essential to assess such awareness and perceptions and make the information available to health education organizations. However, only a few community-based KAP studies towards CL are available in Ethiopia [18, 23, 24], and none existed for Wolaita zone in southern Ethiopia. Therefore, the present study aimed to assess the knowledge, attitude, and practice about CL in that area.

Materials and methods

Study area

The study was conducted in Wolaita zone, southern Ethiopia (Fig 1). The zone is situated at a distance of 330 Km away from Addis Ababa, the Ethiopian capital. The administrative areas in Wolaita zone are structured in *woredas* (districts) and *kebeles* (villages). There are seventeen rural districts and three town administrations in the zone. Wolaita zone is one of Ethiopia's most densely populated zones, with over 342 persons living per square kilometer [25]. The majority of the population in the zone resides in rural districts where the primary livelihood is agriculture [26]. According to the zonal reports, although the existing health facilities are insufficient to

address the service needs of the large population in the zone, there are significant recent improvements regarding health care provision and infrastructure development. However, traditional treatment is still largely practiced for some diseases, mainly among rural communities.

Figure 1. **A location map of the study area (created with ESRI ArcGIS Desktop 10.8).**

Study design

A community-based cross-sectional study was conducted from August to October 2020 in two selected districts of Wolaita zone, Kindo Didaye and Sodo Zuria, based on CL presence information obtained from the local and the zonal health offices. Information about CL presence in the districts was also obtained from the farmer community during reconnaissance surveys conducted in the zone's mid-highland areas. By using the Garmin 72H GPS apparatus, villages having areas above an altitude of 1700 m.a.s.l. were taken: six villages from Kindo Didaye district (Bosa Borto, Bosa Manara, Sime Dolaye, Zebo, Bereda, and Mogisa) and three villages from Sodo Zuria district (Gurumo Woide, Damot Waja, and Dalbo Wogene) were included in this study. The purposive focus on the study areas with the specified altitude was because CL is reported from highland or mid-highland areas in Ethiopia [3, 8, 10, 27].

Sample size and sampling

The sample size was calculated using the single population proportion statistical formula for health studies [28], $n=Z^2 P(1-P)/d^2$,with the following assumption: n = the number of study subjects (household heads), **Z** is a critical value (1.96) at 95% confidence level, P= an anticipated

population proportion assumed to be 50% to obtain a maximum sample size since there was no previously established report on the KAP about CL for the study area, d= the level of precision or margin of error (5%). Therefore, the minimum sample size of the household heads required for this study was 384. After adding a 10% non-response rate, the total sample size was calculated to be 422. To allocate the sample size for each study district (village in the district), the calculated sample size was proportionally divided by the total households present in the CL suspected areas. Every fifth household was systematically selected, and the household head was approached to gather information about the KAP regarding cutaneous leishmaniasis and its prevention.

Data collection procedure

A total of thirty-six data collectors and nine supervisors collected the data. The two-day training was given to data collectors and supervisors before the data collection. Pretesting was done in a neighboring village (not included in the actual data collection) in 5% of the sample size. The data were collected using a pretested structured questionnaire (S1). The questionnaire contained four parts: part one related to the socio-demography of study participants, part two on knowledge regarding CL, part three on practice related to CL prevention and treatment, and part four on attitude towards CL. The questionnaire was prepared in English and then translated into Amharic or the local language, Wolaittatto, as appropriate and back-translated into English to check for consistency.

Scoring knowledge and attitude

Participants' knowledge and attitude were scored according to the method described in other studies [15, 29, 30] with some modifications to adapt to the present study. For each question, a score of 1 point was assigned for answers considered to categorize a respondent as knowledgeable, and a score of 0 point was assigned for answers considered to categorize a respondent not knowledgeable. The knowledge was assessed using a composite variable created from 5-item questions that assessed the knowledge of participants towards CL. For the knowledge questions, the answers considered to categorize a respondent as knowledgeable were: 1) “yes” for “do you know CL?”; 2) “yes” for “do you know zoonosis?”; 3) “by insect bite” for “do you know CL mode of transmission?”; 4) “yes” for “do you know sandflies?” and 5) all except “I don’t know” for “do you know the sign and symptoms of CL?”. The total knowledge scores ranged from 0 to 5. The knowledge scores between 0 and 2 were considered to indicate poor knowledge, while scores between 3 and 5 were considered to indicate good knowledge. The attitude of participants towards CL was assessed by a 5-point Likert scale that ranged from "strongly disagree" to "strongly agree." The composite variable for attitude was created by computing the responses to 5-item questions. The total attitude scores ranged from 0 to 5. The study participants who scored between 0 and 2 were considered to have a "negative," while those who scored between 3 and 5 were considered to have a "positive attitude."

Data analysis

Data analysis was performed with R version 4.1 after entering, cleaning, and organizing the data in Epidata version 4.6.0.2. Descriptive statistics (frequency, percentage and mean) were used to

describe the socio-demographic variables. Categorical variables were presented using frequencies and percentages. Bivariate and multivariate logistic regression analyses were performed to determine the relationship between the participants' knowledge about CL and socio-demographic characteristics. Possible associations were measured using an adjusted odds ratio (AOR) with 95% CI, and a p-value of less than 0.05 was considered statistically significant.

Ethics statement

The study was reviewed and approved by the Institutional Review Board of Wolaita Sodo University under the reference number WSU41/12/1225. Subsequently, Permissions were obtained from Wolaita Zone Health Bureau and the respective District authorities. Written informed consent was obtained from each study participant. All demographic data of the study participants were kept confidential and anonymized before analysis.

Results

Socio‑ demographic profiles of the participants

Of the 422 household heads who participated in this study, 367 (87%) were males, and 55 (13%) were females. The average age of respondents was 43.52 years, with a standard deviation of 10.38 years. The majority (226, 53.6%) of the participants were aged less than or equal to 40 years. Most (368, 87.2%) of the participants were farmers, while the rest were non-farmers. With regard to education, 288 (68.2%) of the participants hadn't had a formal education. The mean family size of the households was 5.51 (SD1.761, range 1-10), with ≤5 individuals living per household in 221 (52.4%) houses (Table 1).

Table 1. Socio-demographic profiles of the household heads.

Variables	Frequency	Percent
Sex		
Male	367	87.0
Female	55	13.0
Age group (year)		
≤40	226	53.6
>40	196	46.4
Occupation		
Farmer	368	87.2
Non-farmers	54	12.8
Status of education		
No formal education*	288	68.2
Formal education	134	31.8
Family size		
≤5	221	52.4
>5	201	47.6

*Refers to no formal school attended.

Knowledge of the participants about CL

CL in the area has local names, "Bolbo" and "Moora," both meaning "disfigure." Most (283, 67.1%) respondents knew CL by its local name though this knowledge varied over the study districts. The majority (369, 87.4%) of respondents hadn't known about zoonosis in general. Though 58 (13.7%) of study participants responded to insects biting as a mode of CL transmission, none knew sandflies. Concerning signs and symptoms of CL, the participants responded that CL disfigures the skin (24.6%), causes lasting wounds (19.7%), mainly occurs on

the face (17.5%), and causes pain (11.4%). Overall, only 80 (19%) participants had good knowledge about CL (Table 2).

Table 2. Knowledge of the participants about cutaneous leishmaniasis in Wolaita zone, south Ethiopia.

Variables	Frequency	%
Know CL		
Yes	283	67.1
No	139	32.9
Know zoonosis		
Yes	53	12.6
No	369	87.4
Know CL mode of transmission		
Contact with an infected person	140	33.2
Contact with infected animals	5	1.2
By insect bite	58	13.7
I don't know	219	51.9
Know sandfly		
Yes	0	0
No	422	100
Know the signs and symptoms of CL		
Mostly occur on the face	74	17.5
Disfigure the skin	104	24.6
Cause lasting wound	83	19.7
Pain	48	11.4
I don't know	113	26.8
Knowledge (overall)		
Good	80	19
Poor	342	81

The attitude of the participants toward CL

One hundred fifty-seven (37.2%) of the participants had a positive attitude towards the treatability of CL. However, 318 (75.4%) of the participants had a negative attitude about the use of modern medication to treat CL. The majority (302, 71.6%) thought CL was a health problem in the area. Although 237 (56.2%) of the respondents had no bad feelings about meeting CL patients, a significant number (185, 43.8%) had a negative attitude about meeting CL patients. One hundred twenty-three (29.1%) study participants thought CL was a stigmatizing disease. The overall 59.5% positive attitude of respondent's about CL was mainly because most perceived CL as a health problem but not a stigmatizing disease (Table 3).

Table 3. **The attitude of the respondents about CL in Wolaita zone, south Ethiopia.**

Variables	Frequency	%
CL is treatable		
Yes	157	37.2
No	265	62.8
CL patients should receive modern medication		
Yes	104	24.6
No	318	75.4
CL is a health problem in the area		
Yes	302	71.6
No	120	28.4
CL is not a stigmatizing disease		
Yes	299	70.9
No	123	29.1
I feel bad when meeting CL patients		
Yes	185	43.8
No	237	56.2
Attitude (overall)		

Positive	251	59.5
Negative	171	40.5

Practice toward CL prevention and treatment

Three hundred twenty-five (77%) of the study participants responded that CL patients preferred to go to the traditional healers seeking treatment. Herbal treatment was the most used (212, 50.2%), followed by burning (100, 25.1%) to treat CL. There was no CL control at a community level. Only 23 (5.5%) respondents used bed nets. The indoor residual spray was also practiced by only 14 (3.3%) of the respondents (Table 4).

Table 4. Practice toward CL treatment and prevention in Wolaita zone, south Ethiopia.

**Variables**	**Frequency**	**%**
Treatment preference		
Traditional healers	325	77
Hospitals/clinics	97	23
Type of medication CL patients receive		
Herbal medicine	212	50.2
Burning	106	25.1
Modern medicine	104	24.6
Community-based CL control		
Yes	0	0
No	422	100
Use bed net		
Yes	23	5.5
No	399	94.5
Use insecticides indoor		
Yes	14	3.3
No	408	96.7

Factors associated with knowledge about CL

Both bivariate and multivariate logistic regression analyses showed that age, sex, and study districts were significantly associated with the overall knowledge of the respondents about CL ($P<0.05$). However, family size, occupation, and educational status were not associated with the respondents' overall knowledge. There were increased odds of having good knowledge among participants who were males (AOR=4.162, 95% CI= 1.386, 12.499), aged 40 years or below (AOR= 2.020, 95% CI= 1.121, 3.639), and those who lived in Kindo Didaye district (AOR=12.379, 95% CI= 4.737, 32.346) than their counterparts (Table 5).

Table 5. Association of knowledge about CL with the socio-demographic profiles of the respondents in Wolaita zone, south Ethiopia. COR: crude odds ratio, AOR: adjusted odds ratio, CI confidence interval.

Variables	Knowledge about CL		COR (95% CI)	AOR (95% CI)	P value for AOR
	Good n (%)	Poor n (%)			
Sex					
Male	76 (20.7)	291 (79.3)	3.330 (1.167, 9.502)*	4.162 (1.386, 12.499)	0.011
Female	4 (7.3)	51 (92.7)	1	1	
Age group (year)					
≤40	58 (25.7)	168 (74.3)	2.731 (1.600, 4.660)*	2.020 (1.121, 3.639)	0.019
>40	22 (11.2)	174 (88.8)	1	1	
Family size					

≤5	35 (15.8)	186 (84.2)	0.652 (0.400, 1.065)	0.658 (0.385, 1.123)	0.125
>5	45 (22.4)	156 (77.6)	1	1	
Occupation					
Non-farmer	15 (27.8)	39 (72.2)	1.793 (0.933, 3.445)	1.233 (0.549, 2.769)	0.612
Farmer	65 (17.7)	303 (82.3)	1	1	
Education					
Formal	32 (23.9)	102 (76.1)	1.569 (0.948, 2.596)	1.728 (0.928, 3.217)	0.085
Non-formal	48 (16.7)	240 (83.3)	1	1	
Study district					
Kindo Didaye	75 (27.7)	196 (72.3)	11.173 (4.407, 8.327)*	12.379 (4.737, 32.346)	0.000
Sodo Zuria	5 (3.3)	146 (96.7)	1	1	

Variables adjusted: Sex, Age group, Family size, Occupation, Education, and Village
*Significant association ($P \leq 0.05$) for COR

Discussion

The present study assessed the knowledge, attitude, and practice (KAP) of the rural community about CL in Kindo Didaye and SodoZuria districts of Wolaita zone, southern Ethiopia. Although CL was perceived as a serious health problem in the study community, we showed a lack of knowledge about and practices toward disease prevention.

The present study participants knew CL by its local name, either "bolbo" or "moora," meaning disfigurement of the exposed parts of the body. Almost all the community groups in Kindo

Didaye district knew CL in the local name, while in Sodo Zuria district, none had the knowledge. The reasons for such differences in usage of CL local name in the present area could be due to the focal nature of disease transmission and the lack of disease-related communication among the community groups. Although most participants recognized CL based on signs/symptoms, they lacked knowledge about its transmission. A considerable proportion (34.4%) of the present participants misunderstood CL transmission as they responded that either "contact with infected people" or "contact with infected animals" was the means of CL transmission. In this study, none of the participants knew sandflies and their role in CL transmission. The lack of knowledge about CL transmission among the current study participants might be due to their low level of education (68.2% didn't attain formal education) and the absence of CL-targeted health education in the area. The current finding is consistent with the results of most community-based KAP studies elsewhere, which reported a better reflection of knowledge on CL symptoms but poor knowledge about its transmission [18, 23, 24, 29]. Contrary to the present finding, better knowledge about CL transmission was reported among some communities [30-32]. In general, the awareness about CL among various communities might depend on the presence of health education programs, socio-economy, and eco-epidemiological factors [30]. The community in CL endemic areas tends to understand better the disease and its transmission [18, 20].

Three-quarters of the respondents of this study considered CL a health problem, but most did not see CL as a stigmatizing disease. The current finding is consistent with the findings from northwest Ethiopia, where most participants believed CL was a serious public health problem [23]. The observed stigma towards CL patients by one-third of respondents in this study can possibly be linked to the wrong perception about CL transmission, seeing it as a contagious

disease that might have existed among the present community groups. CL led to severe psychosocial suffering, including stigma, in some areas where the level of stigma could be much higher than the present finding [33-38]. CL lesions and scars on the face are of considerable social impact due to stigma [39]. Similar explanations were provided based on other findings that a lack of community awareness of CL could lead to increased misunderstandings and wrong beliefs about the disease [11, 40, 41].

About 77% of participants in this study responded that CL patients preferred to go to traditional healers rather than to hospitals or clinics to receive treatment. This finding agrees with the reported higher practices of traditional treatment for CL elsewhere among rural communities of Ethiopia: 68.3% in the Amhara region (northwest Ethiopia) [23], 90% in a district in the Tigray region (north Ethiopia) [18], and 67.6% in Gamo Gofa zone (south Ethiopia) [24]. However, the extent of the use of traditional medication to treat CL could be low in some communities. The differences in the treatment practices of CL among communities in various localities might be due to a lack of community awareness of the available treatment options, accessibility of modern CL treatment centers, the socio-economy, and socio-cultural factors. In localities like the present study area, where modern CL treatment is unavailable, CL patients remain dependent on traditional therapies. The use of herbal preparations and burning CL lesions with red hot metal or clay were the widely practiced traditional methods to treat CL in the present study area. Burning CL lesions was widely practiced in Kndo Didaye district, where most affected community groups live.

The very low prevention practices found in the current study, coupled with the absence of standard CL treatment, implies CL is a highly neglected disease in the area. These might have also resulted in the observed 62.8% negative attitude toward CL treatability. This finding is supported by a study from Yemen [29], where the absence of treatment facilities resulted in an increased negative attitude about CL treatability. Community awareness about the treatability of CL might be high in areas with established leishmaniasis treatment centers [23, 42, 43]. However, the presence of a leishmaniasis treatment center alone may not be sufficient to bring a positive attitude towards CL treatability in endemic areas [24].

In this study, the knowledge about CL was found to be associated with sex, age, and study districts. The male participants had better knowledge as compared to their female counterparts. In a male-dominated rural community [44], males might have better exposure to knowledge about CL. Males were also found more knowledgeable in a KAP study about visceral leishmaniasis in the west Armachiho district, Northwest Ethiopia [45]. However, the association of knowledge about CL with gender was reported differently in other studies in which females had better knowledge about the disease [18, 46]. In the current study, participants aged below or equal to 40 years had better knowledge. This finding agrees with a study done in Bihar, India, where the same age group had a better understanding of CL [15]. Interestingly, this study found that district was the most important factor associated with CL knowledge. Participants of the Kindo Didaye district had better knowledge than the other district. The observed district-wise difference in the Knowledge about CL might be attributed to the eco-epidemiological factors and the associated experiences of the local communities regarding the disease [12, 42].

Limitations

Our study was conducted among communities at higher altitudes. Hence, the findings of this study should not be misinterpreted to the whole population of the zone. The face-to-face interview method of the data collection might have predisposed the respondents to social desirability bias. Nevertheless, we minimized the bias by restricting interviewers not to interview households from the same locality. Despite these limitations, this study presented valuable findings to emphasize health education campaigns and future CL prevention and control plans in the study areas.

Conclusion

This study found a lack of knowledge about and practices toward CL in the study areas. The insufficient knowledge and community practice regarding the infection nature, vector, transmission ways, and treatment and presentation of CL emphasize the need for health education and community mobilization campaign to increase community awareness about the disease in the area. The misconceptions observed about CL treatment also need the urgent establishment of treatment facilities.

List of abbreviations

CL: Cutaneous Leishmaniasis; KAP: Knowledge, Attitude, and Practice; WHO: World Health Organization; GPS: Global Positioning System; NTD: Neglected Tropical Disease

Declarations

Consent for publication

Consent to publish this manuscript from the participants was deemed not applicable since the manuscript does not contain identifying data from any individual person.

Availability of data and material

All relevant data are within the manuscript and its Supporting Information files.

Competing interest

All authors declared that they have no competing interests.

Funding

This work was funded by the Flemish Interuniversity Council (VLIR-UOS, ET2019TEA485A102). The funders had no role in study design, data collection and analysis, decision to publish, or preparation of the manuscript.

Author contributions

Conceptualization: Bereket Alemayehu, Herwig Leirs.

Data curation: Bereket Alemayehu, Chimdesa Adugna, Mihiretu Alemayehu.

Formal analysis: Bereket Alemayehu, Mihiretu Alemayehu.

Funding acquisition: Herwig Leirs.

Investigation: Bereket Alemayehu, Chimdesa Adugna, Tessema Bibo.

Methodology: Bereket Alemayehu, Abraham Getachew, Mihiretu Alemayehu, Aberham Megaze, Herwig Leirs.

Project administration: Bereket Alemayehu, Aberham Megaze, Herwig Leirs.

Resources: Aberham Megaze, Herwig Leirs.

Supervision: Bereket Alemayehu, Aberham Megaze, Herwig Leirs.

Writing – original draft: Bereket Alemayehu.

Writing – review & editing: Bereket Alemayehu, Abraham Getachew, Mihiretu Alemayehu, Chimdesa Adugna, Tessema Bibo, Aberham Megaze, Herwig Leirs.

Acknowledgments

We thank VLIR-UOS, the University of Antwerp, Belgium, and Wolaita Sodo University, Ethiopia, for their technical and administrative support. We also extend our gratitude to Wolaita zone and district administrative bodies, data collectors, data clerks, the local community, and study participants for their cooperation and technical assistance.

References

1. World Health Organization. Cutaneous leishmaniasis control in selected countries of the WHO eastern Mediterranean and African regions. Report of an interregional network meeting Casablanca, Morocco 23–24 June 2014.; 2014.
2. World Health Organization. Global leishmaniasis surveillance, 2017–2018, and first report on 5 additional indicators – Surveillance mondiale de la leishmaniose, 2017-2018, et premier rapport sur 5 indicateurs supplémentaires. Weekly Epidemiological Record. 2020;95(25):265-79

3. Ashford RW, Bray MA, Hutchinson MP, Bray RS. The epidemiology of cutaneous leishmaniasis in Ethiopia. Trans R Soc Trop Med Hyg. 1973;67(4):568-601 DOI: 10.1016/0035-9203(73)90088-6.
4. Alemayehu B, Alemayehu M. Leishmaniasis: A Review on Parasite, Vector and Reservoir Host. Health Sci J. 2017;11(4) DOI: 10.21767/1791-809x.1000519.
5. Leishmaniases WHOECotCot, World Health O. Control of the leishmaniases: report of a meeting of the WHO Expert Commitee on the Control of Leishmaniases, Geneva, 22-26 March 2010. Geneva: World Health Organization; 2010.
6. Deribe K, Meribo K, Gebre T, Hailu A, Ali A, Aseffa A, et al. The burden of neglected tropical diseases in Ethiopia, and opportunities for integrated control and elimination. Parasit Vectors. 2012;5(1):240 DOI: 10.1186/1756-3305-5-240.
7. Hailu A, Di Muccio T, Abebe T, Hunegnaw M, Kager PA, Gramiccia M. Isolation of *Leishmania tropica* from an Ethiopian cutaneous leishmaniasis patient. Trans R Soc Trop Med Hyg. 2006;100(1):53-8 DOI: 10.1016/j.trstmh.2005.04.017.
8. Lemma A, Foster WA, Gemetchu T, Preston PM, Bryceson A, Minter DM. Studies on leishmaniasis in Ethiopia. I. Preliminary investigations into the epidemiology of cutaneous leishmaniasis in the highlands. Ann Trop Med Parasitol. 1969;63(4):455-72
9. Bray RS, Ashford RW, Bray MA. The parasite causing cutaneous leishmaniasis in Ethiopia. Trans R Soc Trop Med Hyg. 1973;67(3):345-8 DOI: 10.1016/0035-9203(73)90111-9.
10. Lemma W, Erenso G, Gadisa E, Balkew M, Gebre-Michael T, Hailu A. A zoonotic focus of cutaneous leishmaniasis in Addis Ababa, Ethiopia. Parasit Vectors. 2009;2(1):60 DOI: 10.1186/1756-3305-2-60.

11. Eshetu B, Mamo H. Cutaneous leishmaniasis in north-central Ethiopia: trend, clinical forms, geographic distribution, and determinants. Trop Med Health. 2020;48

12. Bsrat A, Berhe N, Balkew M, Yohannes M, Teklu T, Gadisa E, et al. Epidemiological study of cutaneous leishmaniasis in Saesie Tsaeda-emba district, eastern Tigray, northern Ethiopia. Parasit Vectors. 2015;8:149 DOI: 10.1186/s13071-015-0758-9.

13. Pareyn M, Van den Bosch E, Girma N, van Houtte N, Van Dongen S, Van der Auwera G, et al. Ecology and seasonality of sandflies and potential reservoirs of cutaneous leishmaniasis in Ochollo, a hotspot in southern Ethiopia. PLoS Negl Trop Dis. 2019;13(8):e0007667 DOI: 10.1371/journal.pntd.0007667.

14. Negera E, Gadisa E, Yamuah L, Engers H, Hussein J, Kuru T, et al. Outbreak of cutaneous leishmaniasis in Silti woreda, Ethiopia: risk factor assessment and causative agent identification. Trans R Soc Trop Med Hyg. 2008;102(9):883-90 DOI: 10.1016/j.trstmh.2008.03.021.

15. J. S D, Gupta AK, Veeri RB, Garapati P, Kumar R, Dhingra S, et al. Knowledge, attitude and practices towards visceral leishmaniasis among HIV patients: A cross-sectional study from Bihar, India. PLoS One. 2021;16(8):e0256239 DOI: 10.1371/journal.pone.0256239.

16. Vázquez ML, Kroeger A, Lipowsky R, Alzate A. [Popular conceptions regarding cutaneous leishmaniasis in Colombia and their applicability in control programs]. Bol Oficina Sanit Panam. 1991;110(5):402-12

17. Ministry of Health of the Federal Democratic Republic of Ethiopia. The Third National Neglected Tropical Diseases Strategic Plan 2021-2025. 2021.

18. Tesfay K, Mardu F, Berhe B, Negash H, Legese H, Adhanom G, et al. Household knowledge, practice and treatment seeking behaviors towards cutaneous leishmaniasis in the endemic rural communities of Ganta- afeshum district, Tigrai, northern Ethiopia, 2019: a cross-sectional study. Trop Dis Travel Med Vaccines. 2021;7(1):19 DOI: 10.1186/s40794-021-00144-4.
19. Nandha B, Srinivasan R, Jambulingam P. Cutaneous leishmaniasis: knowledge, attitude and practices of the inhabitants of the Kani forest tribal settlements of Tiruvananthapuram district, Kerala, India. Health Educ Res. 2014;29(6):1049-57 DOI: 10.1093/her/cyu064.
20. Ahmad S, Obaid MK, Taimur M, Shaheen H, Khan SN, Niaz S, et al. Knowledge, attitude, and practices towards cutaneous leishmaniasis in referral cases with cutaneous lesions: A cross-sectional survey in remote districts of southern Khyber Pakhtunkhwa, Pakistan. PLoS One. 2022;17(5):e0268801 DOI: 10.1371/journal.pone.0268801.
21. Bautista-Gomez MM, Doerfler J, Del Mar Castro M. Barriers to cutaneous leishmaniasis care faced by indigenous communities of rural areas in Colombia: a qualitative study. BMC Infect Dis. 2022;22(1):302 DOI: 10.1186/s12879-022-07204-w.
22. Moussa S AT, Alhudaires KM, Alshammari TS, Alshammari TR, Elgendy AI, et al. . Awareness and behavioral practice of cutaneous leishmaniasis among hail population, kingdom of Saudi Arabia. J Microbiol Exp. 2019;7(2):88-99
23. Tamiru HF, Mashalla YJ, Mohammed R, Tshweneagae GT. Cutaneous leishmaniasis a neglected tropical disease: community knowledge, attitude and practices in an endemic area, Northwest Ethiopia. BMC Infect Dis. 2019;19(1):855 DOI: 10.1186/s12879-019-4506-1.

24. Kebede N, Worku A, Ali A, Animut A, Negash Y, Gebreyes WA, et al. Community knowledge, attitude and practice towards cutaneous leishmaniasis endemic area Ochello, Gamo Gofa Zone, South Ethiopia. Asian Pac J Trop Biomed. 2016;6(7):562-7 DOI: https://doi.org/10.1016/j.apjtb.2016.01.018.

25. Wolaita Zone. Annual Socio-economic and Demographic Reports. Wolaita Sodo, Process DDCaDC; 2015.

26. Cochrane L, Gecho Y. Data on the demographics, education, health and infrastructure: Wolaita Zone, Ethiopia. Data Brief. 2018;21:2095-102 DOI: 10.1016/j.dib.2018.11.063.

27. Pareyn M, Rutten A, Merdekios B, Wedegärtner REM, Girma N, Regelbrugge L, et al. High-resolution habitat suitability model for Phlebotomus pedifer, the vector of cutaneous leishmaniasis in southwestern Ethiopia. Parasit Vectors. 2020;13(1):467 DOI: 10.1186/s13071-020-04336-3.

28. Lwanga SK, Lemeshow S, World Health O. Sample size determination in health studies : a practical manual / S. K. Lwanga and S. Lemeshow. Geneva: World Health Organization; 1991.

29. Alharazi TH, Haouas N, Al-Mekhlafi HM. Knowledge and attitude towards cutaneous leishmaniasis among rural endemic communities in Shara'b district, Taiz, southwestern Yemen. BMC Infect Dis. 2021;21(1):269 DOI: 10.1186/s12879-021-05965-4.

30. Alemu A, Alemu A, Esmael N, Dessie Y, Hamdu K, Mathewos B, et al. Knowledge, attitude and practices related to visceral leishmaniasis among residents in Addis Zemen town,

South Gondar, Northwest Ethiopia. BMC Public Health. 2013;13:382 DOI: 10.1186/1471-2458-13-382.

31. Saleh F, Khan MF, Kabir MR. Awareness of residents about kala-azar and its related practices in two endemic areas of Bangladesh. PLoS One. 2019;14(7):e0219591 DOI: 10.1371/journal.pone.0219591.
32. Sarkari B, Qasem A, Shafaf MR. Knowledge, attitude, and practices related to cutaneous leishmaniasis in an endemic focus of cutaneous leishmaniasis, Southern Iran. Asian Pac J Trop Biomed. 2014;4(7):566-9 DOI: 10.12980/apjtb.4.2014c744.
33. Bailey F, Mondragon-Shem K, Hotez P, Ruiz-Postigo JA, Al-Salem W, Acosta-Serrano A, et al. A new perspective on cutaneous leishmaniasis-Implications for global prevalence and burden of disease estimates. PLoS Negl Trop Dis. 2017;11(8):e0005739 DOI: 10.1371/journal.pntd.0005739.
34. Bennis I, De Brouwere V, Belrhiti Z, Sahibi H, Boelaert M. Psychosocial burden of localised cutaneous Leishmaniasis: a scoping review. BMC Public Health. 2018;18(1):358 DOI: 10.1186/s12889-018-5260-9.
35. Chahed MK, Bellali H, Ben Jemaa S, Bellaj T. Psychological and Psychosocial Consequences of Zoonotic Cutaneous Leishmaniasis among Women in Tunisia: Preliminary Findings from an Exploratory Study. PLoS Negl Trop Dis. 2016;10(10):e0005090 DOI: 10.1371/journal.pntd.0005090.
36. Garapati P, Pal B, Siddiqui NA, Bimal S, Das P, Murti K, et al. Knowledge, stigma, health seeking behaviour and its determinants among patients with post kalaazar dermal leishmaniasis, Bihar, India. PLoS One. 2018;13(9):e0203407 DOI: 10.1371/journal.pone.0203407.

37. Ramdas S, van der Geest S, Schallig HD. Nuancing stigma through ethnography: the case of cutaneous leishmaniasis in Suriname. Soc Sci Med. 2016;151:139-46 DOI: 10.1016/j.socscimed.2015.12.044.

38. Gómez LJ, van Wijk R, van Selm L, Rivera A, Barbosa MC, Parisi S, et al. Stigma, participation restriction and mental distress in patients affected by leprosy, cutaneous leishmaniasis and Chagas disease: a pilot study in two co-endemic regions of eastern Colombia. Trans R Soc Trop Med Hyg. 2020;114(7):476-82 DOI: 10.1093/trstmh/trz132.

39. Yanik M, Gurel MS, Simsek Z, Kati M. The psychological impact of cutaneous leishmaniasis. Clin Exp Dermatol. 2004;29(5):464-7 DOI: 10.1111/j.1365-2230.2004.01605.x.

40. Akram A, Khan HAA, Qadir A, Sabir AM. A Cross-Sectional Survey of Knowledge, Attitude and Practices Related to Cutaneous Leishmaniasis and Sand Flies in Punjab, Pakistan. PLoS One. 2015;10(6):e0130929 DOI: 10.1371/journal.pone.0130929.

41. Okwor I, Uzonna J. Social and Economic Burden of Human Leishmaniasis. Am J Trop Med Hyg. 2016;94(3):489-93 DOI: 10.4269/ajtmh.15-0408.

42. Yohannes M, Abebe Z, Boelee E. Prevalence and environmental determinants of cutaneous leishmaniasis in rural communities in Tigray, northern Ethiopia. PLoS Negl Trop Dis. 2019; 13 (9): e0007722. https://doi.org/10.1371/journal. pntd.0007722

43. Padovese V, Terranova M, Toma L, Barnabas GA, Morrone A. Cutaneous and mucocutaneous leishmaniasis in Tigray, northern Ethiopia: clinical aspects and therapeutic concerns. Trans R Soc Trop Med Hyg. 2009;103(7):707-11 DOI: 10.1016/j.trstmh.2009.02.023.

44. Lailulo YA, Susuman AS, Blignaut R. Correlates of gender characteristics, health and empowerment of women in Ethiopia. BMC women's health. 2015;15:116- DOI: 10.1186/s12905-015-0273-3.

45. Melkamu HT, Beyene AM, Zegeye DT. Knowledge, attitude and practices of the resident community about visceral leishmaniasis in West Armachiho district, Northwest Ethiopia. Heliyon. 2020;6(1):e03152 DOI: 10.1016/j.heliyon.2019.e03152.

46. Dires A, Kumar P, Gedamu S, Yimam W, Ademe S. Knowledge, attitude and prevention measures of students towards cutaneous leishmaniasis in Delanta district, Northeast Ethiopia. Parasite Epidemiol Control. 2022;17:e00241 DOI: https://doi.org/10.1016/j.parepi.2022.e00241.

Supporting information

S1 File. Questionnaire used for the data collection.

S2 File. The dataset of KAP of CL, Wolaita zone, southern Ethiopia (August to October 2020).

Click here to access/download;Figure;Figure 1_Map of the study area.docx

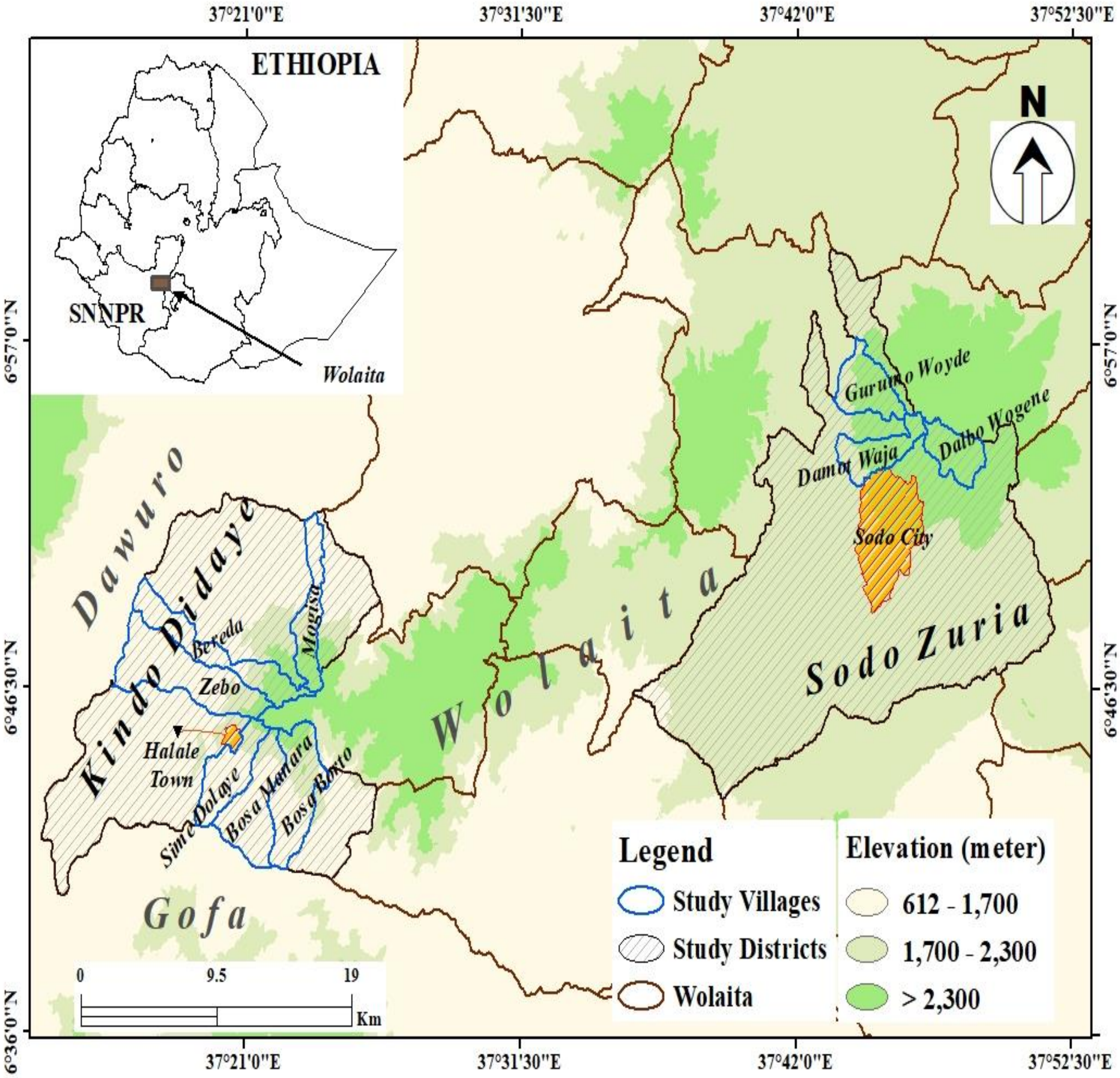

Figure 1. A location map of the study area (created with ESRI ArcGIS Desktop 10.8).

Click here to access/download
Supporting Information
Supporting Information 1_Questionnaire.docx

S2 File. The dataset of KAP of CL, Wolaita zone, southern Ethiopia (August to October 2020)

Click here to access/download

Supporting Information

S2 File. The dataset of KAP of CL_Wolaita zone.sav

Knowledge, attitude, and practice of the rural community about cutaneous leishmaniasis in Wolaita zone, southern Ethiopia

Bereket Alemayehu[1,2]*, Abraham Getachew Kelbore[3], Mihiretu Alemayehu[4], Chimdesa Adugna[1], Tessema Bibo[1], Aberham Megaze[1], Herwig Leirs[2]

[1] Department of Biology, College of Natural and Computational Sciences, Wolaita Sodo University, Wolaita Sodo, Ethiopia

[2] Evolutionary Ecology Group, University of Antwerp, Antwerp, Belgium

[3] Department of Dermatology, College of Health Sciences and Medicine, Wolaita Sodo University, Wolaita Sodo, Ethiopia

[4] School of Public Health, College of Health Sciences and Medicine, Wolaita Sodo University, Wolaita Sodo, Ethiopia

Bereket A: bereketalemayehu@gmail.com (Corresponding author)

Abraham G: kelbore2005@gmail.com

Mihiretu A: mihiretua@gmail.com

Chimdesa A: chimdesaadugna@gmail.com

Tessema B: tessemabibo2015@gmail.com

Aberham M: Abme749@yahoo.com

H Leirs: herwig.leirs@uantwerpen.be

Abstract

Background: Cutaneous leishmaniasis (CL) is a neglected tropical disease that is caused by a *Leishmania* parasite and transmitted by the bite of infected female sandflies. Community awareness is an essential component of disease control and prevention. Therefore, this study

aimed to assess the community's knowledge, attitude, and practice toward CL in Wolaita zone, southern Ethiopia.

Methods: A community-based cross-sectional study design was employed to include 422 study subjects selected using a systematic sampling technique from two districts, Kindo Didaye and Sodo Zuria. A pretested structured questionnaire was used to collect data from the household heads. Bivariate and multivariate logistic regression analyses were performed to determine the relationship between the participants' knowledge about CL and socio-demographic characteristics.

Results: Out of the 422 study participants, only 19% had good knowledge of CL in general. Most (67.1%) of the respondents knew CL by its local name ("bolbo" or "moora") though this knowledge varied highly over the study districts. The majority (86.3%) of respondents did not know how CL is acquired, though they considered CL a health problem. Most (62.8%) respondents believed that CL was an untreatable disease. Most (77%) participants responded that CL patients preferred to go to traditional healers for treatment. Herbal treatment was the most (50.2%) used, followed by burning (25.1%). Knowledge about CL was significantly associated with sex, age, and study districts.

Conclusion: The overall knowledge, attitude, and practice about CL and its prevention in the study area were low. This emphasizes the need to implement health education and awareness campaign to reduce the risk of CL infection. Policymakers and stakeholders should also give due attention to the prevention and treatment of CL in the study area.

Keywords: Cutaneous leishmaniasis, *Leishmania*, Knowledge, Practice, Wolaita zone, Ethiopia

Introduction

Cutaneous leishmaniasis (CL) is a neglected tropical disease (NTD) and is caused by a protozoan parasite of the genus *Leishmania*. It is a vector-borne disease transmitted by the bite of infected female sandflies of various species [1]. The disease is the most common form of leishmaniasis, which causes skin lesions on the exposed parts of the body, leaving life-long scars and serious disability or stigma. WHO estimated that 600,000 to 1 million new cases of CL occur worldwide annually, though this might be an underestimation due to misdiagnosis and lack of reporting. About 95% of CL cases occurred in the Americas, the Mediterranean Basin, the Middle East, Central Asia, and the Sub-Saharan region. In 2018, of the 200 countries and territories reported to WHO to have leishmaniasis, 44% were considered endemic for CL [2]. In endemic countries, the transmission cycle of CL is very complex, involving various species of parasites, sandfly vectors, and reservoir hosts [3, 4].

Ethiopia is one of the world's CL endemic countries, with an estimated incidence of 20,000 to 50,000 cases annually [5, 6]. Almost all CL infection in Ethiopia is caused by a *L. aethiopica* parasite. Infection with this parasite can result in a mild localized CL to a more severe form, diffused CL [7-9]. The Ethiopian CL is reported to be zoonotic, involving hyraxes as reservoir hosts, and the transmission to the human host is effected through blood-feeding *Phlebotomine* sandflies [3, 8-10].

Studies have reported the magnitude of CL and its ecology in Ethiopia until recently [3, 11-14]. However, the disease continues to be a public health problem in the country. There is a lack of knowledge about CL infection among communities in the country, which hinders community

participation in disease prevention and control [15, 16]. The national master plan of NTDs of Ethiopia has prioritized community engagement as one of the core elements to achieve the national goals of preventing and controlling NTDs, including CL [17].

Rural communities bear the highest disease burden [18-20]. The lack of health facilities and inaccessibility of appropriate treatments worsen the situation [21]. Due to a lack of awareness, there are also misconceptions about CL among remote communities [22]. Community-based knowledge, attitude, and practice (KAP) studies are therefore essential to assess such awareness and perceptions and make the information available to health education organizations. However, only a few community-based KAP studies towards CL are available in Ethiopia [18, 23, 24], and none existed for Wolaita zone in southern Ethiopia. Therefore, the present study aimed to assess the knowledge, attitude, and practice about CL in that area.

Materials and methods

Study area

The study was conducted in Wolaita zone, southern Ethiopia (Fig 1). The zone is situated at a distance of 330 Km away from Addis Ababa, the Ethiopian capital. The administrative areas in Wolaita zone are structured in *woredas* (districts) and *kebeles* (villages). There are seventeen rural districts and three town administrations in the zone. Wolaita zone is one of Ethiopia's most densely populated zones, with over 342 persons living per square kilometer [25]. The majority of the population in the zone resides in rural districts where the primary livelihood is agriculture [26]. According to the zonal reports, although the existing health facilities are insufficient to

address the service needs of the large population in the zone, there are significant recent improvements regarding health care provision and infrastructure development. However, traditional treatment is still largely practiced for some diseases, mainly among rural communities.

Figure 1. **A location map of the study area (created with ESRI ArcGIS Desktop 10.8).**

Study design

A community-based cross-sectional study was conducted from August to October 2020 in two selected districts of Wolaita zone, Kindo Didaye and Sodo Zuria, based on CL presence information obtained from the local and the zonal health offices. Information about CL presence in the districts was also obtained from the farmer community during reconnaissance surveys conducted in the zone's mid-highland areas. By using the Garmin 72H GPS apparatus, villages having areas above an altitude of 1700 m.a.s.l. were taken: six villages from Kindo Didaye district (Bosa Borto, Bosa Manara, Sime Dolaye, Zebo, Bereda, and Mogisa) and three villages from Sodo Zuria district (Gurumo Woide, Damot Waja, and Dalbo Wogene) were included in this study. The purposive focus on the study areas with the specified altitude was because CL is reported from highland or mid-highland areas in Ethiopia [3, 8, 10, 27].

Sample size and sampling

The sample size was calculated using the single population proportion statistical formula for health studies [28], $n=Z^2 P(1-P)/d^2$,with the following assumption: n = the number of study subjects (household heads), **Z** is a critical value (1.96) at 95% confidence level, P= an anticipated

population proportion assumed to be 50% to obtain a maximum sample size since there was no previously established report on the KAP about CL for the study area, d= the level of precision or margin of error (5%). Therefore, the minimum sample size of the household heads required for this study was 384. After adding a 10% non-response rate, the total sample size was calculated to be 422. To allocate the sample size for each study district (village in the district), the calculated sample size was proportionally divided by the total households present in the CL suspected areas. Every fifth household was systematically selected, and the household head was approached to gather information about the KAP regarding cutaneous leishmaniasis and its prevention.

Data collection procedure

A total of thirty-six data collectors and nine supervisors collected the data. The two-day training was given to data collectors and supervisors before the data collection. Pretesting was done in a neighboring village (not included in the actual data collection) in 5% of the sample size. The data were collected using a pretested structured questionnaire (S1). The questionnaire contained four parts: part one related to the socio-demography of study participants, part two on knowledge regarding CL, part three on practice related to CL prevention and treatment, and part four on attitude towards CL. The questionnaire was prepared in English and then translated into Amharic or the local language, Wolaittatto, as appropriate and back-translated into English to check for consistency.

Scoring knowledge and attitude

Participants' knowledge and attitude were scored according to the method described in other studies [15, 29, 30] with some modifications to adapt to the present study. For each question, a score of 1 point was assigned for answers considered to categorize a respondent as knowledgeable, and a score of 0 point was assigned for answers considered to categorize a respondent not knowledgeable. The knowledge was assessed using a composite variable created from 5-item questions that assessed the knowledge of participants towards CL. For the knowledge questions, the answers considered to categorize a respondent as knowledgeable were: 1) "yes" for "do you know CL?"; 2) "yes" for "do you know zoonosis?"; 3) "by insect bite" for "do you know CL mode of transmission?"; 4) "yes" for "do you know sandflies?" and 5) all except "I don't know" for "do you know the sign and symptoms of CL?". The total knowledge scores ranged from 0 to 5. The knowledge scores between 0 and 2 were considered to indicate poor knowledge, while scores between 3 and 5 were considered to indicate good knowledge. The attitude of participants towards CL was assessed by a 5-point Likert scale that ranged from "strongly disagree" to "strongly agree." The composite variable for attitude was created by computing the responses to 5-item questions. The total attitude scores ranged from 0 to 5. The study participants who scored between 0 and 2 were considered to have a "negative," while those who scored between 3 and 5 were considered to have a "positive attitude."

Data analysis

Data analysis was performed with R version 4.1 after entering, cleaning, and organizing the data in Epidata version 4.6.0.2. Descriptive statistics (frequency, percentage and mean) were used to

describe the socio-demographic variables. Categorical variables were presented using frequencies and percentages. Bivariate and multivariate logistic regression analyses were performed to determine the relationship between the participants' knowledge about CL and socio-demographic characteristics. Possible associations were measured using an adjusted odds ratio (AOR) with 95% CI, and a p-value of less than 0.05 was considered statistically significant.

Ethics statement

The study was reviewed and approved by the Institutional Review Board of Wolaita Sodo University under the reference number WSU41/12/1225. Subsequently, Permissions were obtained from Wolaita Zone Health Bureau and the respective District authorities. Written informed consent was obtained from each study participant. All demographic data of the study participants were kept confidential and anonymized before analysis.

Results

Socio‐ demographic profiles of the participants

Of the 422 household heads who participated in this study, 367 (87%) were males, and 55 (13%) were females. The average age of respondents was 43.52 years, with a standard deviation of 10.38 years. The majority (226, 53.6%) of the participants were aged less than or equal to 40 years. Most (368, 87.2%) of the participants were farmers, while the rest were non-farmers. With regard to education, 288 (68.2%) of the participants hadn't had a formal education. The mean family size of the households was 5.51 (SD1.761, range 1-10), with ≤5 individuals living per household in 221 (52.4%) houses (Table 1).

Table 1. Socio-demographic profiles of the household heads.

Variables	Frequency	Percent
Sex		
Male	367	87.0
Female	55	13.0
Age group (year)		
≤40	226	53.6
>40	196	46.4
Occupation		
Farmer	368	87.2
Non-farmers	54	12.8
Status of education		
No formal education*	288	68.2
Formal education	134	31.8
Family size		
≤5	221	52.4
>5	201	47.6

*Refers to no formal school attended.

Knowledge of the participants about CL

CL in the area has local names, "Bolbo" and "Moora," both meaning "disfigure." Most (283, 67.1%) respondents knew CL by its local name though this knowledge varied over the study districts. The majority (369, 87.4%) of respondents hadn't known about zoonosis in general. Though 58 (13.7%) of study participants responded to insects biting as a mode of CL transmission, none knew sandflies. Concerning signs and symptoms of CL, the participants responded that CL disfigures the skin (24.6%), causes lasting wounds (19.7%), mainly occurs on

the face (17.5%), and causes pain (11.4%). Overall, only 80 (19%) participants had good knowledge about CL (Table 2).

Table 2. Knowledge of the participants about cutaneous leishmaniasis in Wolaita zone, south Ethiopia.

**Variables**	**Frequency**	**%**
Know CL		
Yes	283	67.1
No	139	32.9
Know zoonosis		
Yes	53	12.6
No	369	87.4
Know CL mode of transmission		
Contact with an infected person	140	33.2
Contact with infected animals	5	1.2
By insect bite	58	13.7
I don't know	219	51.9
Know sandfly		
Yes	0	0
No	422	100
Know the signs and symptoms of CL		
Mostly occur on the face	74	17.5
Disfigure the skin	104	24.6
Cause lasting wound	83	19.7
Pain	48	11.4
I don't know	113	26.8
Knowledge (overall)		
Good	80	19
Poor	342	81

The attitude of the participants toward CL

One hundred fifty-seven (37.2%) of the participants had a positive attitude towards the treatability of CL. However, 318 (75.4%) of the participants had a negative attitude about the use of modern medication to treat CL. The majority (302, 71.6%) thought CL was a health problem in the area. Although 237 (56.2%) of the respondents had no bad feelings about meeting CL patients, a significant number (185, 43.8%) had a negative attitude about meeting CL patients. One hundred twenty-three (29.1%) study participants thought CL was a stigmatizing disease. The overall 59.5% positive attitude of respondent's about CL was mainly because most perceived CL as a health problem but not a stigmatizing disease (Table 3).

Table 3. The attitude of the respondents about CL in Wolaita zone, south Ethiopia.

Variables	Frequency	%
CL is treatable		
Yes	157	37.2
No	265	62.8
CL patients should receive modern medication		
Yes	104	24.6
No	318	75.4
CL is a health problem in the area		
Yes	302	71.6
No	120	28.4
CL is not a stigmatizing disease		
Yes	299	70.9
No	123	29.1
I feel bad when meeting CL patients		
Yes	185	43.8
No	237	56.2
Attitude (overall)		

Positive	251	59.5
Negative	171	40.5

Practice toward CL prevention and treatment

Three hundred twenty-five (77%) of the study participants responded that CL patients preferred to go to the traditional healers seeking treatment. Herbal treatment was the most used (212, 50.2%), followed by burning (100, 25.1%) to treat CL. There was no CL control at a community level. Only 23 (5.5%) respondents used bed nets. The indoor residual spray was also practiced by only 14 (3.3%) of the respondents (Table 4).

Table 4. Practice toward CL treatment and prevention in Wolaita zone, south Ethiopia.

**Variables**	**Frequency**	**%**
Treatment preference		
Traditional healers	325	77
Hospitals/clinics	97	23
Type of medication CL patients receive		
Herbal medicine	212	50.2
Burning	106	25.1
Modern medicine	104	24.6
Community-based CL control		
Yes	0	0
No	422	100
Use bed net		
Yes	23	5.5
No	399	94.5
Use insecticides indoor		
Yes	14	3.3
No	408	96.7

Factors associated with knowledge about CL

Both bivariate and multivariate logistic regression analyses showed that age, sex, and study districts were significantly associated with the overall knowledge of the respondents about CL ($P<0.05$). However, family size, occupation, and educational status were not associated with the respondents' overall knowledge. There were increased odds of having good knowledge among participants who were males (AOR=4.162, 95% CI= 1.386, 12.499), aged 40 years or below (AOR= 2.020, 95% CI= 1.121, 3.639), and those who lived in Kindo Didaye district (AOR=12.379, 95% CI= 4.737, 32.346) than their counterparts (Table 5).

Table 5. Association of knowledge about CL with the socio-demographic profiles of the respondents in Wolaita zone, south Ethiopia. COR: crude odds ratio, AOR: adjusted odds ratio, CI confidence interval.

Variables	Knowledge about CL		COR (95% CI)	AOR (95% CI)	P value for AOR
	Good n (%)	Poor n (%)			
Sex					
Male	76 (20.7)	291 (79.3)	3.330 (1.167, 9.502)*	4.162 (1.386, 12.499)	0.011
Female	4 (7.3)	51 (92.7)	1	1	
Age group (year)					
≤40	58 (25.7)	168 (74.3)	2.731 (1.600, 4.660)*	2.020 (1.121, 3.639)	0.019
>40	22 (11.2)	174 (88.8)	1	1	
Family size					

≤5	35 (15.8)	186 (84.2)	0.652 (0.400, 1.065)	0.658 (0.385, 1.123)	0.125
>5	45 (22.4)	156 (77.6)	1	1	
Occupation					
Non-farmer	15 (27.8)	39 (72.2)	1.793 (0.933, 3.445)	1.233 (0.549, 2.769)	0.612
Farmer	65 (17.7)	303 (82.3)	1	1	
Education					
Formal	32 (23.9)	102 (76.1)	1.569 (0.948, 2.596)	1.728 (0.928, 3.217)	0.085
Non-formal	48 (16.7)	240 (83.3)	1	1	
Study district					
Kindo Didaye	75 (27.7)	196 (72.3)	11.173 (4.407, 8.327)*	12.379 (4.737, 32.346)	0.000
Sodo Zuria	5 (3.3)	146 (96.7)	1	1	

Variables adjusted: Sex, Age group, Family size, Occupation, Education, and Village
*Significant association ($P \leq 0.05$) for COR

Discussion

The present study assessed the knowledge, attitude, and practice (KAP) of the rural community about CL in Kindo Didaye and SodoZuria districts of Wolaita zone, southern Ethiopia. Although CL was perceived as a serious health problem in the study community, we showed a lack of knowledge about and practices toward disease prevention.

The present study participants knew CL by its local name, either "bolbo" or "moora," meaning disfigurement of the exposed parts of the body. Almost all the community groups in Kindo

Didaye district knew CL in the local name, while in Sodo Zuria district, none had the knowledge. The reasons for such differences in usage of CL local name in the present area could be due to the focal nature of disease transmission and the lack of disease-related communication among the community groups. Although most participants recognized CL based on signs/symptoms, they lacked knowledge about its transmission. A considerable proportion (34.4%) of the present participants misunderstood CL transmission as they responded that either "contact with infected people" or "contact with infected animals" was the means of CL transmission. In this study, none of the participants knew sandflies and their role in CL transmission. The lack of knowledge about CL transmission among the current study participants might be due to their low level of education (68.2% didn't attain formal education) and the absence of CL-targeted health education in the area. The current finding is consistent with the results of most community-based KAP studies elsewhere, which reported a better reflection of knowledge on CL symptoms but poor knowledge about its transmission [18, 23, 24, 29]. Contrary to the present finding, better knowledge about CL transmission was reported among some communities [30-32]. In general, the awareness about CL among various communities might depend on the presence of health education programs, socio-economy, and eco-epidemiological factors [30]. The community in CL endemic areas tends to understand better the disease and its transmission [18, 20].

Three-quarters of the respondents of this study considered CL a health problem, but most did not see CL as a stigmatizing disease. The current finding is consistent with the findings from northwest Ethiopia, where most participants believed CL was a serious public health problem [23]. The observed stigma towards CL patients by one-third of respondents in this study can possibly be linked to the wrong perception about CL transmission, seeing it as a contagious

disease that might have existed among the present community groups. CL led to severe psychosocial suffering, including stigma, in some areas where the level of stigma could be much higher than the present finding [33-38]. CL lesions and scars on the face are of considerable social impact due to stigma [39]. Similar explanations were provided based on other findings that a lack of community awareness of CL could lead to increased misunderstandings and wrong beliefs about the disease [11, 40, 41].

About 77% of participants in this study responded that CL patients preferred to go to traditional healers rather than to hospitals or clinics to receive treatment. This finding agrees with the reported higher practices of traditional treatment for CL elsewhere among rural communities of Ethiopia: 68.3% in the Amhara region (northwest Ethiopia) [23], 90% in a district in the Tigray region (north Ethiopia) [18], and 67.6% in Gamo Gofa zone (south Ethiopia) [24]. However, the extent of the use of traditional medication to treat CL could be low in some communities. The differences in the treatment practices of CL among communities in various localities might be due to a lack of community awareness of the available treatment options, accessibility of modern CL treatment centers, the socio-economy, and socio-cultural factors. In localities like the present study area, where modern CL treatment is unavailable, CL patients remain dependent on traditional therapies. The use of herbal preparations and burning CL lesions with red hot metal or clay were the widely practiced traditional methods to treat CL in the present study area. Burning CL lesions was widely practiced in Kndo Didaye district, where most affected community groups live.

The very low prevention practices found in the current study, coupled with the absence of standard CL treatment, implies CL is a highly neglected disease in the area. These might have also resulted in the observed 62.8% negative attitude toward CL treatability. This finding is supported by a study from Yemen [29], where the absence of treatment facilities resulted in an increased negative attitude about CL treatability. Community awareness about the treatability of CL might be high in areas with established leishmaniasis treatment centers [23, 42, 43]. However, the presence of a leishmaniasis treatment center alone may not be sufficient to bring a positive attitude towards CL treatability in endemic areas [24].

In this study, the knowledge about CL was found to be associated with sex, age, and study districts. The male participants had better knowledge as compared to their female counterparts. In a male-dominated rural community [44], males might have better exposure to knowledge about CL. Males were also found more knowledgeable in a KAP study about visceral leishmaniasis in the west Armachiho district, Northwest Ethiopia [45]. However, the association of knowledge about CL with gender was reported differently in other studies in which females had better knowledge about the disease [18, 46]. In the current study, participants aged below or equal to 40 years had better knowledge. This finding agrees with a study done in Bihar, India, where the same age group had a better understanding of CL [15]. Interestingly, this study found that district was the most important factor associated with CL knowledge. Participants of the Kindo Didaye district had better knowledge than the other district. The observed district-wise difference in the Knowledge about CL might be attributed to the eco-epidemiological factors and the associated experiences of the local communities regarding the disease [12, 42].

Limitations

Our study was conducted among communities at higher altitudes. Hence, the findings of this study should not be misinterpreted to the whole population of the zone. The face-to-face interview method of the data collection might have predisposed the respondents to social desirability bias. Nevertheless, we minimized the bias by restricting interviewers not to interview households from the same locality. Despite these limitations, this study presented valuable findings to emphasize health education campaigns and future CL prevention and control plans in the study areas.

Conclusion

This study found a lack of knowledge about and practices toward CL in the study areas. The insufficient knowledge and community practice regarding the infection nature, vector, transmission ways, and treatment and presentation of CL emphasize the need for health education and community mobilization campaign to increase community awareness about the disease in the area. The misconceptions observed about CL treatment also need the urgent establishment of treatment facilities.

List of abbreviations

CL: Cutaneous Leishmaniasis; KAP: Knowledge, Attitude, and Practice; WHO: World Health Organization; GPS: Global Positioning System; NTD: Neglected Tropical Disease

Declarations

Consent for publication

Consent to publish this manuscript from the participants was deemed not applicable since the manuscript does not contain identifying data from any individual person.

Availability of data and material

All relevant data are within the manuscript and its Supporting Information files.

Competing interest

All authors declared that they have no competing interests.

Funding

This work was funded by the Flemish Interuniversity Council (VLIR-UOS, ET2019TEA485A102). The funders had no role in study design, data collection and analysis, decision to publish, or preparation of the manuscript.

Author contributions

Conceptualization: Bereket Alemayehu, Herwig Leirs.

Data curation: Bereket Alemayehu, Chimdesa Adugna, Mihiretu Alemayehu.

Formal analysis: Bereket Alemayehu, Mihiretu Alemayehu.

Funding acquisition: Herwig Leirs.

Investigation: Bereket Alemayehu, Chimdesa Adugna, Tessema Bibo.

Methodology: Bereket Alemayehu, Abraham Getachew, Mihiretu Alemayehu, Aberham Megaze, Herwig Leirs.

Project administration: Bereket Alemayehu, Aberham Megaze, Herwig Leirs.

Resources: Aberham Megaze, Herwig Leirs.

Supervision: Bereket Alemayehu, Aberham Megaze, Herwig Leirs.

Writing – original draft: Bereket Alemayehu.

Writing – review & editing: Bereket Alemayehu, Abraham Getachew, Mihiretu Alemayehu, Chimdesa Adugna, Tessema Bibo, Aberham Megaze, Herwig Leirs.

Acknowledgments

We thank VLIR-UOS, the University of Antwerp, Belgium, and Wolaita Sodo University, Ethiopia, for their technical and administrative support. We also extend our gratitude to Wolaita zone and district administrative bodies, data collectors, data clerks, the local community, and study participants for their cooperation and technical assistance.

References

1. World Health Organization. Cutaneous leishmaniasis control in selected countries of the WHO eastern Mediterranean and African regions. Report of an interregional network meeting Casablanca, Morocco 23–24 June 2014.; 2014.
2. World Health Organization. Global leishmaniasis surveillance, 2017–2018, and first report on 5 additional indicators – Surveillance mondiale de la leishmaniose, 2017-2018, et premier rapport sur 5 indicateurs supplémentaires. Weekly Epidemiological Record. 2020;95(25):265-79

3. Ashford RW, Bray MA, Hutchinson MP, Bray RS. The epidemiology of cutaneous leishmaniasis in Ethiopia. Trans R Soc Trop Med Hyg. 1973;67(4):568-601 DOI: 10.1016/0035-9203(73)90088-6.
4. Alemayehu B, Alemayehu M. Leishmaniasis: A Review on Parasite, Vector and Reservoir Host. Health Sci J. 2017;11(4) DOI: 10.21767/1791-809x.1000519.
5. Leishmaniases WHOECotCot, World Health O. Control of the leishmaniases: report of a meeting of the WHO Expert Commitee on the Control of Leishmaniases, Geneva, 22-26 March 2010. Geneva: World Health Organization; 2010.
6. Deribe K, Meribo K, Gebre T, Hailu A, Ali A, Aseffa A, et al. The burden of neglected tropical diseases in Ethiopia, and opportunities for integrated control and elimination. Parasit Vectors. 2012;5(1):240 DOI: 10.1186/1756-3305-5-240.
7. Hailu A, Di Muccio T, Abebe T, Hunegnaw M, Kager PA, Gramiccia M. Isolation of *Leishmania tropica* from an Ethiopian cutaneous leishmaniasis patient. Trans R Soc Trop Med Hyg. 2006;100(1):53-8 DOI: 10.1016/j.trstmh.2005.04.017.
8. Lemma A, Foster WA, Gemetchu T, Preston PM, Bryceson A, Minter DM. Studies on leishmaniasis in Ethiopia. I. Preliminary investigations into the epidemiology of cutaneous leishmaniasis in the highlands. Ann Trop Med Parasitol. 1969;63(4):455-72
9. Bray RS, Ashford RW, Bray MA. The parasite causing cutaneous leishmaniasis in Ethiopia. Trans R Soc Trop Med Hyg. 1973;67(3):345-8 DOI: 10.1016/0035-9203(73)90111-9.
10. Lemma W, Erenso G, Gadisa E, Balkew M, Gebre-Michael T, Hailu A. A zoonotic focus of cutaneous leishmaniasis in Addis Ababa, Ethiopia. Parasit Vectors. 2009;2(1):60 DOI: 10.1186/1756-3305-2-60.

11. Eshetu B, Mamo H. Cutaneous leishmaniasis in north-central Ethiopia: trend, clinical forms, geographic distribution, and determinants. Trop Med Health. 2020;48

12. Bsrat A, Berhe N, Balkew M, Yohannes M, Teklu T, Gadisa E, et al. Epidemiological study of cutaneous leishmaniasis in Saesie Tsaeda-emba district, eastern Tigray, northern Ethiopia. Parasit Vectors. 2015;8:149 DOI: 10.1186/s13071-015-0758-9.

13. Pareyn M, Van den Bosch E, Girma N, van Houtte N, Van Dongen S, Van der Auwera G, et al. Ecology and seasonality of sandflies and potential reservoirs of cutaneous leishmaniasis in Ochollo, a hotspot in southern Ethiopia. PLoS Negl Trop Dis. 2019;13(8):e0007667 DOI: 10.1371/journal.pntd.0007667.

14. Negera E, Gadisa E, Yamuah L, Engers H, Hussein J, Kuru T, et al. Outbreak of cutaneous leishmaniasis in Silti woreda, Ethiopia: risk factor assessment and causative agent identification. Trans R Soc Trop Med Hyg. 2008;102(9):883-90 DOI: 10.1016/j.trstmh.2008.03.021.

15. J. S D, Gupta AK, Veeri RB, Garapati P, Kumar R, Dhingra S, et al. Knowledge, attitude and practices towards visceral leishmaniasis among HIV patients: A cross-sectional study from Bihar, India. PLoS One. 2021;16(8):e0256239 DOI: 10.1371/journal.pone.0256239.

16. Vázquez ML, Kroeger A, Lipowsky R, Alzate A. [Popular conceptions regarding cutaneous leishmaniasis in Colombia and their applicability in control programs]. Bol Oficina Sanit Panam. 1991;110(5):402-12

17. Ministry of Health of the Federal Democratic Republic of Ethiopia. The Third National Neglected Tropical Diseases Strategic Plan 2021-2025. 2021.

18. Tesfay K, Mardu F, Berhe B, Negash H, Legese H, Adhanom G, et al. Household knowledge, practice and treatment seeking behaviors towards cutaneous leishmaniasis in the endemic rural communities of Ganta- afeshum district, Tigrai, northern Ethiopia, 2019: a cross-sectional study. Trop Dis Travel Med Vaccines. 2021;7(1):19 DOI: 10.1186/s40794-021-00144-4.

19. Nandha B, Srinivasan R, Jambulingam P. Cutaneous leishmaniasis: knowledge, attitude and practices of the inhabitants of the Kani forest tribal settlements of Tiruvananthapuram district, Kerala, India. Health Educ Res. 2014;29(6):1049-57 DOI: 10.1093/her/cyu064.

20. Ahmad S, Obaid MK, Taimur M, Shaheen H, Khan SN, Niaz S, et al. Knowledge, attitude, and practices towards cutaneous leishmaniasis in referral cases with cutaneous lesions: A cross-sectional survey in remote districts of southern Khyber Pakhtunkhwa, Pakistan. PLoS One. 2022;17(5):e0268801 DOI: 10.1371/journal.pone.0268801.

21. Bautista-Gomez MM, Doerfler J, Del Mar Castro M. Barriers to cutaneous leishmaniasis care faced by indigenous communities of rural areas in Colombia: a qualitative study. BMC Infect Dis. 2022;22(1):302 DOI: 10.1186/s12879-022-07204-w.

22. Moussa S AT, Alhudaires KM, Alshammari TS, Alshammari TR, Elgendy AI, et al. . Awareness and behavioral practice of cutaneous leishmaniasis among hail population, kingdom of Saudi Arabia. J Microbiol Exp. 2019;7(2):88-99

23. Tamiru HF, Mashalla YJ, Mohammed R, Tshweneagae GT. Cutaneous leishmaniasis a neglected tropical disease: community knowledge, attitude and practices in an endemic area, Northwest Ethiopia. BMC Infect Dis. 2019;19(1):855 DOI: 10.1186/s12879-019-4506-1.

24. Kebede N, Worku A, Ali A, Animut A, Negash Y, Gebreyes WA, et al. Community knowledge, attitude and practice towards cutaneous leishmaniasis endemic area Ochello, Gamo Gofa Zone, South Ethiopia. Asian Pac J Trop Biomed. 2016;6(7):562-7 DOI: https://doi.org/10.1016/j.apjtb.2016.01.018.
25. Wolaita Zone. Annual Socio-economic and Demographic Reports. Wolaita Sodo, Process DDCaDC; 2015.
26. Cochrane L, Gecho Y. Data on the demographics, education, health and infrastructure: Wolaita Zone, Ethiopia. Data Brief. 2018;21:2095-102 DOI: 10.1016/j.dib.2018.11.063.
27. Pareyn M, Rutten A, Merdekios B, Wedegärtner REM, Girma N, Regelbrugge L, et al. High-resolution habitat suitability model for Phlebotomus pedifer, the vector of cutaneous leishmaniasis in southwestern Ethiopia. Parasit Vectors. 2020;13(1):467 DOI: 10.1186/s13071-020-04336-3.
28. Lwanga SK, Lemeshow S, World Health O. Sample size determination in health studies : a practical manual / S. K. Lwanga and S. Lemeshow. Geneva: World Health Organization; 1991.
29. Alharazi TH, Haouas N, Al-Mekhlafi HM. Knowledge and attitude towards cutaneous leishmaniasis among rural endemic communities in Shara'b district, Taiz, southwestern Yemen. BMC Infect Dis. 2021;21(1):269 DOI: 10.1186/s12879-021-05965-4.
30. Alemu A, Alemu A, Esmael N, Dessie Y, Hamdu K, Mathewos B, et al. Knowledge, attitude and practices related to visceral leishmaniasis among residents in Addis Zemen town,

South Gondar, Northwest Ethiopia. BMC Public Health. 2013;13:382 DOI: 10.1186/1471-2458-13-382.

31. Saleh F, Khan MF, Kabir MR. Awareness of residents about kala-azar and its related practices in two endemic areas of Bangladesh. PLoS One. 2019;14(7):e0219591 DOI: 10.1371/journal.pone.0219591.

32. Sarkari B, Qasem A, Shafaf MR. Knowledge, attitude, and practices related to cutaneous leishmaniasis in an endemic focus of cutaneous leishmaniasis, Southern Iran. Asian Pac J Trop Biomed. 2014;4(7):566-9 DOI: 10.12980/apjtb.4.2014c744.

33. Bailey F, Mondragon-Shem K, Hotez P, Ruiz-Postigo JA, Al-Salem W, Acosta-Serrano A, et al. A new perspective on cutaneous leishmaniasis-Implications for global prevalence and burden of disease estimates. PLoS Negl Trop Dis. 2017;11(8):e0005739 DOI: 10.1371/journal.pntd.0005739.

34. Bennis I, De Brouwere V, Belrhiti Z, Sahibi H, Boelaert M. Psychosocial burden of localised cutaneous Leishmaniasis: a scoping review. BMC Public Health. 2018;18(1):358 DOI: 10.1186/s12889-018-5260-9.

35. Chahed MK, Bellali H, Ben Jemaa S, Bellaj T. Psychological and Psychosocial Consequences of Zoonotic Cutaneous Leishmaniasis among Women in Tunisia: Preliminary Findings from an Exploratory Study. PLoS Negl Trop Dis. 2016;10(10):e0005090 DOI: 10.1371/journal.pntd.0005090.

36. Garapati P, Pal B, Siddiqui NA, Bimal S, Das P, Murti K, et al. Knowledge, stigma, health seeking behaviour and its determinants among patients with post kalaazar dermal leishmaniasis, Bihar, India. PLoS One. 2018;13(9):e0203407 DOI: 10.1371/journal.pone.0203407.

37. Ramdas S, van der Geest S, Schallig HD. Nuancing stigma through ethnography: the case of cutaneous leishmaniasis in Suriname. Soc Sci Med. 2016;151:139-46 DOI: 10.1016/j.socscimed.2015.12.044.

38. Gómez LJ, van Wijk R, van Selm L, Rivera A, Barbosa MC, Parisi S, et al. Stigma, participation restriction and mental distress in patients affected by leprosy, cutaneous leishmaniasis and Chagas disease: a pilot study in two co-endemic regions of eastern Colombia. Trans R Soc Trop Med Hyg. 2020;114(7):476-82 DOI: 10.1093/trstmh/trz132.

39. Yanik M, Gurel MS, Simsek Z, Kati M. The psychological impact of cutaneous leishmaniasis. Clin Exp Dermatol. 2004;29(5):464-7 DOI: 10.1111/j.1365-2230.2004.01605.x.

40. Akram A, Khan HAA, Qadir A, Sabir AM. A Cross-Sectional Survey of Knowledge, Attitude and Practices Related to Cutaneous Leishmaniasis and Sand Flies in Punjab, Pakistan. PLoS One. 2015;10(6):e0130929 DOI: 10.1371/journal.pone.0130929.

41. Okwor I, Uzonna J. Social and Economic Burden of Human Leishmaniasis. Am J Trop Med Hyg. 2016;94(3):489-93 DOI: 10.4269/ajtmh.15-0408.

42. Yohannes M, Abebe Z, Boelee E. Prevalence and environmental determinants of cutaneous leishmaniasis in rural communities in Tigray, northern Ethiopia. PLoS Negl Trop Dis. 2019; 13 (9): e0007722. https://doi.org/10.1371/journal. pntd.0007722

43. Padovese V, Terranova M, Toma L, Barnabas GA, Morrone A. Cutaneous and mucocutaneous leishmaniasis in Tigray, northern Ethiopia: clinical aspects and therapeutic concerns. Trans R Soc Trop Med Hyg. 2009;103(7):707-11 DOI: 10.1016/j.trstmh.2009.02.023.

44. Lailulo YA, Susuman AS, Blignaut R. Correlates of gender characteristics, health and empowerment of women in Ethiopia. BMC women's health. 2015;15:116- DOI: 10.1186/s12905-015-0273-3.

45. Melkamu HT, Beyene AM, Zegeye DT. Knowledge, attitude and practices of the resident community about visceral leishmaniasis in West Armachiho district, Northwest Ethiopia. Heliyon. 2020;6(1):e03152 DOI: 10.1016/j.heliyon.2019.e03152.

46. Dires A, Kumar P, Gedamu S, Yimam W, Ademe S. Knowledge, attitude and prevention measures of students towards cutaneous leishmaniasis in Delanta district, Northeast Ethiopia. Parasite Epidemiol Control. 2022;17:e00241 DOI: https://doi.org/10.1016/j.parepi.2022.e00241.

Supporting information

S1 File. Questionnaire used for the data collection.

S2 File. The dataset of KAP of CL, Wolaita zone, southern Ethiopia (August to October 2020).

Response to Reviewers (PLOS ONE)

Manuscript Number: PONE-D-22-15781

Knowledge, attitude, and practice of the rural community about cutaneous leishmaniasis in Wolaita zone, southern Ethiopia

We thank the editor and the reviewers for their invaluable and constructive comments and suggestions. We could further improve our paper based on the provided comments and suggestions. We made the requested changes in the manuscript (yellow colored), and please find our point-by-point responses below. We hope the amendments below address the comments and suggestions forwarded by the academic editor and the reviewers.

Academic Editor's Comments:

Comments: Journal Requirements: When submitting your revision, we need you to address these additional requirements.

Comment #1. Please ensure that your manuscript meets PLOS ONE's style requirements, including those for file naming.

Response: Thank you for reminding us to maintain the PLOS ONE's style requirements. We referred to the PLOS ONE style templates and addressed the issues regarding the requirements and styles of the journal in the revised manuscript.

Comment #2. We note that you have provided funding information that is not currently declared in your Funding Statement. However, funding information should not appear in the Acknowledgments section or other areas of your manuscript. We will only publish funding information present in the Funding Statement section of the online submission form. Please remove any funding-related text from the manuscript and let us know how you would like to update your Funding Statement. Currently, your Funding Statement reads as follows: This work was funded by the Flemish Interuniversity Council (VLIR-UOS, ET2019TEA485A102). The funders had no role in study design, data collection, and analysis, decision to publish, or preparation of the manuscript.

Please include your amended statements within your cover letter; we will change the online submission form on your behalf.

Response: We removed the funding-related texts in the revised version of the manuscript. The funding information was declared/entered as a funding statement in the "Financial Disclosure" section of the online submission form during the initial submission (we checked its presence in

the pdf generated during the submission). If it is absent from the online submission form, we are thankful to the Editor in advance for entering the same funding statement (without updating/amendment) in the system on our behalf.

Comment #3. We note that you have stated that you will provide repository information for your data at acceptance. Should your manuscript be accepted for publication, we will hold it until you provide the relevant accession numbers or DOIs necessary to access your data. If you wish to make changes to your Data Availability statement, please describe these changes in your cover letter and we will update your Data Availability statement to reflect the information you provide.

Response: We are thankful to the Editor for the information. The repository information was put in by mistake. All relevant data are within the manuscript and its Supporting Information files. We will describe the changes to our Data Availability statement in our cover letter, and we thank you in advance for you will update the statement.

Comment #4. Your ethics statement should only appear in the Methods section of your manuscript. If your ethics statement is written in any section besides the Methods, please delete it from any other section.

Response: The comment is accepted, and now the ethics statement appears only in our manuscript's "Method" section.

Comment #5. We note that Figure 1 in your submission contain map/satellite images which may be copyrighted. All PLOS content is published under the Creative Commons Attribution License (CC BY 4.0), which means that the manuscript, images, and Supporting Information files will be freely available online, and any third party is permitted to access, download, copy, distribute, and use these materials in any way, even commercially, with proper attribution. For these reasons, we cannot publish previously copyrighted maps or satellite images created using proprietary data, such as Google software (Google Maps, Street View, and Earth). For more information, see our copyright guidelines: http://journals.plos.org/plosone/s/licenses-and-copyright.

Response: We appreciate the Editor for the concern. The map in our manuscript is original (not previously copyrighted).

Comment #6. Please include captions for your Supporting Information files at the end of your manuscript, and update any in-text citations to match accordingly. Please see our Supporting Information guidelines for more information: http://journals.plos.org/plosone/s/supporting-information.

Response: We included the caption of the Supporting Information at the end of our manuscript.

Reviewers' Comments:

Reviewer #1

Comment #1. I reviewed a manuscript entitled “Knowledge, attitude, and practice of the rural community about cutaneous leishmaniasis in Wolaita zone, southern Ethiopia”. The work is intended to investigate the knowledge, attitude, and practice (KAP) of the community toward CL in the Wolaita zone, southern Ethiopia. The study concludes that the overall knowledge, attitude, and practice about CL and its prevention in the study area were low. The study is interesting and such types of studies provide the baseline information for disease control and management in endemic areas. The study has merit and should be considered for publication; however, I recommend minor revision before its publication in PLoS One. Factors associated with knowledge about CL are among the key results of the study, however, these results are not mentioned in the abstract. I would suggest to please mention these results in the abstract.

Response: We appreciate the reviewer for giving us these important comments. The key results of the knowledge about CL and its associated factors are now mentioned in in the “Abstract” of the revised manuscript.

Comment #2. In the discussion, the authors should also include some studies from other countries to support the current findings. This will add further diversity to the findings and will make the study of more international interest. Some examples of such studies are suggested below.

1. Doe ED, Egyir-Yawson A, Kwakye-Nuako G. Knowledge, attitude, and practices related to cutaneous leishmaniasis in endemic communities in the Volta region of Ghana. Int J Health Sci. 2019; 7(1):12.

2. Moussa S, Alshammari T, Alhudaires K, Alshammari T, Alshammari T, Elgendy A. Awareness, and behavioural practice of cutaneous leishmaniasis among hail population, Kingdom of Saudi Arabia. J Microbiol Exp. 2019; 7(2):88–9.

3. Ahmad S, Obaid MK, Taimur M, Shaheen H, Khan SN, Niaz S, Ali R, Haleem S. Knowledge, attitude, and practices towards cutaneous leishmaniasis in referral cases with cutaneous lesions: A cross-sectional survey in remote districts of southern Khyber Pakhtunkhwa, Pakistan. PLoS One. 2022 26;17(5): e0268801. doi: 10.1371/journal.pone.0268801. PMID: 35617283; PMCID: PMC9135282.

4. Zeinali M, Mohebali M, Mahmoudi M, Hassanpour GR, Shirza di MR. Study on knowledge, attitude, and practice of health workers of East Azerbaijan, Ilam and Khorasan Razavi provinces about leishmaniasis during 2015–2016: a comparative study before and after the intervention. Arch Clin Infect Dis. 2019; 14(1): e64282

Response: We are also thankful to the reviewer for suggesting supportive articles. We have incorporated these now in the Discussion.

Comment #3. The references should be revised as per the PLoS One author's guidelines https://journals.plos.org/plosone/s/submission-guidelines.

In some references the journal name is not abbreviated.

Response: We checked the reference guidelines and abbreviated the journal names now.

Reviewer #2

The article is relatively well written and is about CL in an area from which CL reports are scarce. However, I have a few issues with the article that should be improved before it can be considered for publication

MAJOR COMMENTS

The rationale for this study is not well explained in the introduction at all. The introduction is very general and does not explain what is already known and what is still unclear and why this is relevant. The introduction should be shortened, all the general stuff about CL in other countries can be removed, the different subspecies and the parts about eco-epidemiology are not related to this manuscript and are distracting. The introduction should be rewritten focused on why a KAP study is needed, what is already known and what is not yet known, also why a KAP would help in control. If there are already several KAP studies done in Ethiopia, it should be very clear why another one is needed. Simply repeating something in a new district is not enough to sell a study.

Response: We appreciate the reviewer for the comments and suggestions. We shortened the introduction and made it more KAP-focused and informative about the scarcity of KAP studies.

The sampling of the study subjects is not clear to me and should be elaborated or mentioned in the discussion as a limitation. Why were household heads chosen?

Response: The household heads were chosen since we believed that they have the best knowledge about the household's characteristics and their community in general. Evidence also indicated that most of the CL KAP-related surveys were conducted by including a similar study population (Tesfaye *et al*., 2021 (BMC Tropical Diseases, Travel Medicine and Vaccines); Sarkari *et al.*, 2014 (Asian Pacific Journal of Tropical Biomedicine); Alemu *et al.*, 2013 (BMC Public Health)).

Is this really the right population to study KAP? Is this a representative population for your community survey? Are they generally the patients who will be affected by CL or is CL more common in children and females in this area?

Response: Yes, definitely, our study population is the right population to study KAP about the present disease. In our opinion, the KAP study shouldn't only be conducted among patients or victims. KAP studies should also be done in the general community in disease-affected areas.

We should also see how the community members/household heads (not only the patients) understand the disease and practice preventions/controls related to the disease. The household heads (as community members) serve as the source of information even to assess how the treatment for a particular disease (CL in our case) is viewed/perceived by the community or by the patients. Choosing a study population from the community depends on the research question.

Yes, the study population is representative for our KAP survey. Identifying the prevalence of CL among respondents was not the objective of this survey, nor was it to determine which community group was affected more. For the population representativeness in the community survey, we used a widely accepted statistical approach to determine the sample size. (mentioned under sample size and sampling). We took a 50% proportion (p) to obtain the maximum sample size since we lacked a similar study. This estimation is beneficial to get the maximum sample size for the study at the calculated 5% margin of error. Then we randomly sampled the household heads systematically. There are other studies that used the same statistical approach: Saleh *et al*., 2019; Dires *et al*., 2022, Tesfaye *et al*., 2021, and others.

The questions related to treatment practices may be better asked to patients who are actually suffering from CL rather than household heads not affected by CL.

Response: We appreciate the reviewer's opinion. We do not deny that actually applied treatment practices may be better asked of the patient. However, in our survey, we assessed the community's view/perception about CL treatment and how they also see the treatment practices of CL patients. However, we had no aim to specifically assess the patients' KAP. If we view it in terms of control and prevention goals, community participation is valuable. The KAP of the community about CL treatment is as equally important as the KAP of CL patients. Community participation is prioritized as a key element in the national health strategic plan of Ethiopia to prevent/control neglected tropical disease (NTD), including CL (Ministry of Health of Ethiopia. The 3rd national NTD strategic plan, 2021).

The authors categorize all the outcome variables into Good and Bad, but I don't really see the added value of this. What is the value of this classification? Is this a standardized tool that they were using? It would be useful to add the questionnaire as a supplementary file. To me it would be more useful to simply describe the knowledge, attitude and practices rather than look at associated factors, especially because the sampling setup doesn't lead to a representative population.

Response: We have not categorized all outcome variables into "Good" and "Bad". Rather, we used "Good", "Poor", "favorable", unfavorable", etc as measures for the composite variables. We used a standard tool to create composite variables that other similar studies had applied (Alharazi et al., 2021 (BMC Infectious Diseases); Alemu et al., 2013 (BMC Public Health); J.S *et al*., 2021 (PLoS One)). We mentioned how the composite variables were created and how terms of the output variables were used in the "Scoring knowledge and attitude" in the method section. Such approaches are familiarly used when KAP studies are analyzed. We also cited sources that were used as references for our method in the revised manuscript (page 7, line 146). The "associated factor" analysis seems valuable for our paper as the result indicated that there were associations between the participants' knowledge about CL and the socio-demographic

variables. This part of the manuscript would particularly support stakeholders, decision/policymakers, and other interested bodies in taking evidence-based actions/interventions to enhance the CL-related knowledge of the population. A very interesting point this analysis showed is the magnitude of the differences in CL knowledge between the study districts. It pointed out that not just the geographically distant places but the nearby community groups might also have differences in CL knowledge. We interpreted this in the discussion section.

We already submitted a questionnaire as a supplementary file during the submission process, and this is in-text cited (S1) in the "Data collection procedure" section of the method in our manuscript (page 6, line 138). We used a standard questionnaire adapted from other similar studies (J.S et al., 2021 (PLoS One), Ahmad et al., 2022 (PLoS One)).

In the response above, we explained the representation of the study population to our survey. We hope that the population representation is clarified to the reviewer. Since identifying associated factors is one of our objectives, we believe that removing/omitting associated factors would compromise the aim and implication of the study.

Missing data is not clearly described. Per variable, the number of missing values should be given. Additionally, the number of people who refused to participate should be mentioned.

Response: There was no missing data in our study. There were also no participants who refused to participate in the study.

What does it mean to have good knowledge of CL? Which variables are classified as ' correct' and which are ' wrong' ? My worry is that these things are not black and white, not all CL lesions are painful or disfiguring, how do you assess whether participants know a sandfly or not or whether they know CL or not? These things should be more clearly described.

Response: We appreciate the questions raised by the reviewer and addressed them in the revised manuscript (page 7). We have already described that the community knows CL by local names (because the disease has existed there since long, according to the inhabitants' information). During the preparation phases (before data collection) of the research, we also obtained information from the district and zonal health offices that CL has existed in the area since long ago, and the local community calls it "bolbo/moora" (page 5, line 110). In our prevalence study (which will be published later elsewhere), the molecular data confirmed that sampled lesions that locally were identified as "bolbo" or "moora", were effectively CL lesions. Variables were defined prior to the data collection, and their operational definitions were stated in the methods section of the manuscript (scoring).

How the attitude part of the questionnaire was done is unclear. It is mentioned that likert scales were done, ranging from 0 (strongly disagree) to 5 (strongly agree), but that the total attitude score ranged from 0-5. I would assume a maximum score of 25 would be possible if a patient strongly agreed with all statements? The attitude questions are classified as having unfavourable attitude and favourable attitude, but it is unclear what is really meant by this as the term is vague and seems to be more about treatment than the disease itself. Why is considered negative if a

disease is not treatable or a health problem or not? I would suggest to simply describe attitudes and not classify them as good and bad.

Response: We appreciate the reviewer's suggestions/comments. Our analysis is the other way (and widely used) of the reviewer's suggestion. We created a composite variable with the scales mentioned. A respondent was assigned a maximum score of 5 when he/she strongly agreed with all questions and 0 when strongly disagreed with all questions. We created the composite variable in this way. The scoring methods were presented in the manuscript (page 7, line 156). The composite variables better indicated the cumulative KAP responses. Then we also pointed out our view in that it sounds better if we measure the degree of attitude (as good, bad, favorable, unfavorable, etc) than by giving a simple description. There are previously published reports that used similar methods and analyses (Devipriya et al., 2021 (PLoS One), Ahmad et al., 2022 (PLoS One), and Kebede et al., 2016 (Asian Pacific Journal of Tropical Biomedicine)). We hope that the revisions made to the paper shape it in a way the reviewer can accept.

Similarly, for practices I would advise to simply describe, rather than calling it good or bad practices. Why is it good or bad practice for CL prevention to live with domestic animals? Why is it poor practice to go to traditional healers?

Response: CL is a treatable disease. However, the attitude of the respondents was assessed, and a portion of them perceived it as "not treatable". When we assessed the attitude towards the use of modern medication (which we consider a "good practice"), the attitude was not in favor of modern medication.

In addition, I think asking about CL treatment and prevention practices is more appropriately asked to CL patients rather than community members.

Response: Thanks again for the opinion. We explained our choice to opt for a community-based approach already mentioned above.

The authors do a regression analysis to look at factors associated with CL knowledge. I would advise them to remove this, as it doesn't really make sense. Especially since the population is not representative, the classification into good or poor is not strong, and the rationale of it is not clear.

Response: We addressed the population representativeness in our survey in the response above. The regression analysis in relation to the CL knowledge showed significant/interesting associations, particularly with the districts that can be helpful to evidence-based action/intervention. Its importance was also mentioned by the other reviewer, and we were commented to include it in the abstract. So we decided to keep the analysis.

The discussion is a bit long, and there is some repetition. I would advise the authors to make it more concise. Important things to add are a section on limitations and the relevance of this manuscript.

Response: We appreciate the reviewer for the valuable suggestions/comments. We accepted the comment and revised the discussion; we also included the study limitations in the manuscript.

MINOR COMMENTS (also see PDF)

Were there any CL patients among the recruited participants?

Response: Yes, but we did not make any analysis in terms of CL patients, as our study population was the household heads in the general community.

It is not clear what the sample size is based on, is that knowledge? Attitude? Practices? This should be specified

Response: we haven't separately calculated the sample size for knowledge, attitude, and practices. We calculated the largest sample size that could be obtained with the formula (mentioned above and in the MS).

Something more about the burden of CL in this area should be explained, what is the endemicity of the two districts and the villages that were sampled?

Response: It needs a separate study to report the burden/ endemicity level of the districts and the villages. We investigated that question in a separate paper that will be published later.

Classification of variables is not clearly explained. Why was age cutoff of 40 used? How was no formal education classified? Why was a family size of 5 used as a cutoff?

Response: The sociodemographic variables were classified in order to provide logical comparisons based on the nature of our study population. The age category is because the participants were adults (household heads) who had higher ages. To clarify "formal education", reference (table footnote) is included under the table in the revised manuscript. The family size cutoff is reasonably classified as the average size of the households in the study area is nearly 5.

Reviewer #3

Thank you for the opportunity to review the manuscript titled "Knowledge, attitude, and practice of the rural community about cutaneous leishmaniasis in Wolaita zone, southern Ethiopia". I would like to appreciate the authors' effort to undertake this valuable task. Based on my point of view I raised the following points that I believed might improve the manuscript. This work plans to assess the community's knowledge, attitude, and practice about cutaneous leishmaniasis in Wolaita zone, southern Ethiopia. This kind of researches provides baseline information for future studies. However, there are some concerns which when addressed hope to improve the quality of the manuscript. Find my specific comments below.

Comment #1- Line 42, "What plants were used in herbal treatment?

Response: We appreciate the reviewer for the question. In this work, we didn't study the type of plants used to treat the disease. Because, the herbal medicinal plants are known by the traditional healers (individual practitioners) and not by community members. So, in this particular study, we didn't approach the traditional healers to obtain information about the plants used for treatment.

Comment #2- Line 54, "Cutaneous leishmaniasis…. For the first time, write it completely and then use its abbreviation (CL)

Response: We accepted the comment.

Comment #3- Line 63, "In 2020, about 85% of new….. The reference related to this sentence is for 2010.

Response: We accepted the comment and addressed it in the revised manuscript.

Comment #4- Line 65, East African countries, including Ethiopia, are…… There is a duplicate with the last paragraph of the introduction.

Response: We accepted the comment; we revised the paragraphs.

Comment #5- Table 1: Instead of level of education I would recommend use of "years of education". Reads might not be familiar with Ethiopian system of education.

Response: We are thankful to the reviewer for the recommendation. However, the data we collected for this variable (education) was not in the "year/s" of education of the respondents. If the reviewer is concerned about the technicality of the word "level", we used "status" instead in the revised manuscript to contextualize the word.

Comment #6- Did you have any missing data among the variables?

Response: No.

Comment #7- I suggest the authors put raw data in an online open access repository for preserve and share their research outputs.

Response: We accepted the suggestion. We are happy to do that. For the ethical concern, we will remove any personal identifying information from the raw data.

Comment #8- Line 251, " perhaps??? In the south of Ethiopia, there are similar studies, please write clearly whether there is a similar study in your studied area.

Response: There is no similar study in our study area. We revised the paragraph.

References

9- Italicize the scientific names (Leishmania tropica in REF 13)

10- Journal names should appear in abbreviation.

11- No journal name should start with "the"

12- Books need city.

Response: We appreciate the reviewer for the comments. We addressed the comments in the revised manuscript.
