# Supplementary material for: Knowledge, attitude, and practice of the rural community about cutaneous leishmaniasis in Wolaita zone, southern Ethiopia
Source: PLoS One. 2023 Mar 28;18(3):e0283582. doi: 10.1371/journal.pone.0283582 (PMC10047512; doi:10.1371/journal.pone.0283582)
Supplement: Attachment — Submitted filename: Response to Reviewers.docx [file pone.0283582.s004.docx]

Response to Reviewers (PLOS ONE)

Manuscript Number: PONE-D-22-15781

**Knowledge, attitude, and practice of the rural community about cutaneous leishmaniasis in Wolaita zone, southern Ethiopia**

We thank the editor and the reviewers for their invaluable and constructive comments and suggestions. We could further improve our paper based on the provided comments and suggestions. We made the requested changes in the manuscript (yellow colored), and please find our point-by-point responses below. We hope the amendments below address the comments and suggestions forwarded by the academic editor and the reviewers.

**Academic Editor's Comments:**

Comments: Journal Requirements: When submitting your revision, we need you to address these additional requirements.

Comment #1. Please ensure that your manuscript meets PLOS ONE's style requirements, including those for file naming.

Response: Thank you for reminding us to maintain the PLOS ONE’s style requirements. We referred to the PLOS ONE style templates and addressed the issues regarding the requirements and styles of the journal in the revised manuscript.

Comment #2. We note that you have provided funding information that is not currently declared in your Funding Statement. However, funding information should not appear in the Acknowledgments section or other areas of your manuscript. We will only publish funding information present in the Funding Statement section of the online submission form. 
Please remove any funding-related text from the manuscript and let us know how you would like to update your Funding Statement. Currently, your Funding Statement reads as follows: 
 This work was funded by the Flemish Interuniversity Council (VLIR-UOS, ET2019TEA485A102). The funders had no role in study design, data collection, and analysis, decision to publish, or preparation of the manuscript.

Please include your amended statements within your cover letter; we will change the online submission form on your behalf.

Response: We removed the funding-related texts in the revised version of the manuscript. The funding information was declared/entered as a funding statement in the “Financial Disclosure” section of the online submission form during the initial submission (we checked its presence in the pdf generated during the submission). If it is absent from the online submission form, we are thankful to the Editor in advance for entering the same funding statement (without updating/amendment) in the system on our behalf.

Comment #3. We note that you have stated that you will provide repository information for your data at acceptance. Should your manuscript be accepted for publication, we will hold it until you provide the relevant accession numbers or DOIs necessary to access your data. If you wish to make changes to your Data Availability statement, please describe these changes in your cover letter and we will update your Data Availability statement to reflect the information you provide.

Response: We are thankful to the Editor for the information. The repository information was put in by mistake. All relevant data are within the manuscript and its Supporting Information files. We will describe the changes to our Data Availability statement in our cover letter, and we thank you in advance for you will update the statement.

Comment #4. Your ethics statement should only appear in the Methods section of your manuscript. If your ethics statement is written in any section besides the Methods, please delete it from any other section.

Response: The comment is accepted, and now the ethics statement appears only in our manuscript’s “Method” section.

Comment #5. We note that Figure 1 in your submission contain map/satellite images which may be copyrighted. All PLOS content is published under the Creative Commons Attribution License (CC BY 4.0), which means that the manuscript, images, and Supporting Information files will be freely available online, and any third party is permitted to access, download, copy, distribute, and use these materials in any way, even commercially, with proper attribution. For these reasons, we cannot publish previously copyrighted maps or satellite images created using proprietary data, such as Google software (Google Maps, Street View, and Earth). For more information, see our copyright guidelines: <http://journals.plos.org/plosone/s/licenses-and-copyright>.

Response: We appreciate the Editor for the concern. The map in our manuscript is original (not previously copyrighted).

Comment #6. Please include captions for your Supporting Information files at the end of your manuscript, and update any in-text citations to match accordingly. Please see our Supporting Information guidelines for more information: http://journals.plos.org/plosone/s/supporting-information.

Response: We included the caption of the Supporting Information at the end of our manuscript.

**Reviewers' Comments:**

**Reviewer #1**

Comment #1. I reviewed a manuscript entitled “Knowledge, attitude, and practice of the rural community about cutaneous leishmaniasis in Wolaita zone, southern Ethiopia”. The work is intended to investigate the knowledge, attitude, and practice (KAP) of the community toward CL in the Wolaita zone, southern Ethiopia. The study concludes that the overall knowledge, attitude, and practice about CL and its prevention in the study area were low. The study is interesting and such types of studies provide the baseline information for disease control and management in endemic areas. The study has merit and should be considered for publication; however, I recommend minor revision before its publication in PLoS One. Factors associated with knowledge about CL are among the key results of the study, however, these results are not mentioned in the abstract. I would suggest to please mention these results in the abstract.

Response: We appreciate the reviewer for giving us these important comments. The key results of the knowledge about CL and its associated factors are now mentioned in in the “Abstract” of the revised manuscript.

Comment #2. In the discussion, the authors should also include some studies from other countries to support the current findings. This will add further diversity to the findings and will make the study of more international interest. Some examples of such studies are suggested below.

1. Doe ED, Egyir-Yawson A, Kwakye-Nuako G. Knowledge, attitude, and practices related to cutaneous leishmaniasis in endemic communities in the Volta region of Ghana. Int J Health Sci. 2019; 7(1):12.

2. Moussa S, Alshammari T, Alhudaires K, Alshammari T, Alshammari T, Elgendy A. Awareness, and behavioural practice of cutaneous leishmaniasis among hail population, Kingdom of Saudi Arabia. J Microbiol Exp. 2019; 7(2):88–9.

3. Ahmad S, Obaid MK, Taimur M, Shaheen H, Khan SN, Niaz S, Ali R, Haleem S. Knowledge, attitude, and practices towards cutaneous leishmaniasis in referral cases with cutaneous lesions: A cross-sectional survey in remote districts of southern Khyber Pakhtunkhwa, Pakistan. PLoS One. 2022 26;17(5): e0268801. doi: 10.1371/journal.pone.0268801. PMID: 35617283; PMCID: PMC9135282.

4. Zeinali M, Mohebali M, Mahmoudi M, Hassanpour GR, Shirza di MR. Study on knowledge, attitude, and practice of health workers of East Azerbaijan, Ilam and Khorasan Razavi provinces about leishmaniasis during 2015–2016: a comparative study before and after the intervention. Arch Clin Infect Dis. 2019; 14(1): e64282

Response: We are also thankful to the reviewer for suggesting supportive articles. We have incorporated these now in the Discussion.

Comment #3. The references should be revised as per the PLoS One author’s guidelines <https://journals.plos.org/plosone/s/submission-guidelines>.

In some references the journal name is not abbreviated.

Response: We checked the reference guidelines and abbreviated the journal names now.

**Reviewer #2**

The article is relatively well written and is about CL in an area from which CL reports are scarce. However, I have a few issues with the article that should be improved before it can be considered for publication

MAJOR COMMENTS

The rationale for this study is not well explained in the introduction at all. The introduction is very general and does not explain what is already known and what is still unclear and why this is relevant. The introduction should be shortened, all the general stuff about CL in other countries can be removed, the different subspecies and the parts about eco-epidemiology are not related to this manuscript and are distracting. The introduction should be rewritten focused on why a KAP study is needed, what is already known and what is not yet known, also why a KAP would help in control. If there are already several KAP studies done in Ethiopia, it should be very clear why another one is needed. Simply repeating something in a new district is not enough to sell a study.

Response: We appreciate the reviewer for the comments and suggestions. We shortened the introduction and made it more KAP-focused and informative about the scarcity of KAP studies.

The sampling of the study subjects is not clear to me and should be elaborated or mentioned in the discussion as a limitation. Why were household heads chosen?

Response: The household heads were chosen since we believed that they have the best knowledge about the household’s characteristics and their community in general. Evidence also indicated that most of the CL KAP-related surveys were conducted by including a similar study population (Tesfaye *et al*., 2021 (BMC Tropical Diseases, Travel Medicine and Vaccines); Sarkari *et al.,* 2014 (Asian Pacific Journal of Tropical Biomedicine); Alemu *et al.,* 2013 (BMC Public Health)).

Is this really the right population to study KAP? Is this a representative population for your community survey? Are they generally the patients who will be affected by CL or is CL more common in children and females in this area?

Response: Yes, definitely, our study population is the right population to study KAP about the present disease. In our opinion, the KAP study shouldn’t only be conducted among patients or victims. KAP studies should also be done in the general community in disease-affected areas. We should also see how the community members/household heads (not only the patients) understand the disease and practice preventions/controls related to the disease. The household heads (as community members) serve as the source of information even to assess how the treatment for a particular disease (CL in our case) is viewed/perceived by the community or by the patients. Choosing a study population from the community depends on the research question.

Yes, the study population is representative for our KAP survey. Identifying the prevalence of CL among respondents was not the objective of this survey, nor was it to determine which community group was affected more. For the population representativeness in the community survey, we used a widely accepted statistical approach to determine the sample size. (mentioned under sample size and sampling). We took a 50% proportion (p) to obtain the maximum sample size since we lacked a similar study. This estimation is beneficial to get the maximum sample size for the study at the calculated 5% margin of error. Then we randomly sampled the household heads systematically. There are other studies that used the same statistical approach: Saleh *et al*., 2019; Dires *et al*., 2022, Tesfaye *et al*., 2021, and others.

The questions related to treatment practices may be better asked to patients who are actually suffering from CL rather than household heads not affected by CL.

Response: We appreciate the reviewer’s opinion. We do not deny that actually applied treatment practices may be better asked of the patient. However, in our survey, we assessed the community’s view/perception about CL treatment and how they also see the treatment practices of CL patients. However, we had no aim to specifically assess the patients’ KAP. If we view it in terms of control and prevention goals, community participation is valuable. The KAP of the community about CL treatment is as equally important as the KAP of CL patients. Community participation is prioritized as a key element in the national health strategic plan of Ethiopia to prevent/control neglected tropical disease (NTD), including CL (Ministry of Health of Ethiopia. The 3^rd^ national NTD strategic plan, 2021).

The authors categorize all the outcome variables into Good and Bad, but I don’t really see the added value of this. What is the value of this classification? Is this a standardized tool that they were using? It would be useful to add the questionnaire as a supplementary file.
To me it would be more useful to simply describe the knowledge, attitude and practices rather than look at associated factors, especially because the sampling setup doesn’t lead to a representative population.

Response: We have not categorized all outcome variables into “Good” and “Bad”. Rather, we used “Good”, “Poor”, “favorable”, unfavorable”, etc as measures for the composite variables. We used a standard tool to create composite variables that other similar studies had applied (Alharazi et al., 2021 (BMC Infectious Diseases); Alemu et al., 2013 (BMC Public Health); J.S *et al*., 2021 (PLoS One)). We mentioned how the composite variables were created and how terms of the output variables were used in the “Scoring knowledge and attitude” in the method section. Such approaches are familiarly used when KAP studies are analyzed. We also cited sources that were used as references for our method in the revised manuscript (page 7, line 146). The “associated factor” analysis seems valuable for our paper as the result indicated that there were associations between the participants' knowledge about CL and the socio-demographic variables. This part of the manuscript would particularly support stakeholders, decision/policymakers, and other interested bodies in taking evidence-based actions/interventions to enhance the CL-related knowledge of the population. A very interesting point this analysis showed is the magnitude of the differences in CL knowledge between the study districts. It pointed out that not just the geographically distant places but the nearby community groups might also have differences in CL knowledge. We interpreted this in the discussion section.

We already submitted a questionnaire as a supplementary file during the submission process, and this is in-text cited (S1) in the “Data collection procedure” section of the method in our manuscript (page 6, line 138). We used a standard questionnaire adapted from other similar studies (J.S et al., 2021 (PLoS One), Ahmad et al., 2022 (PLoS One)).

In the response above, we explained the representation of the study population to our survey. We hope that the population representation is clarified to the reviewer. Since identifying associated factors is one of our objectives, we believe that removing/omitting associated factors would compromise the aim and implication of the study.

Missing data is not clearly described. Per variable, the number of missing values should be given. Additionally, the number of people who refused to participate should be mentioned.

Response: There was no missing data in our study. There were also no participants who refused to participate in the study.

What does it mean to have good knowledge of CL? Which variables are classified as ‘ correct’ and which are ‘ wrong’ ? My worry is that these things are not black and white, not all CL lesions are painful or disfiguring, how do you assess whether participants know a sandfly or not or whether they know CL or not? These things should be more clearly described.

Response: We appreciate the questions raised by the reviewer and addressed them in the revised manuscript (page 7). We have already described that the community knows CL by local names (because the disease has existed there since long, according to the inhabitants’ information). During the preparation phases (before data collection) of the research, we also obtained information from the district and zonal health offices that CL has existed in the area since long ago, and the local community calls it “bolbo/moora” (page 5, line 110). In our prevalence study (which will be published later elsewhere), the molecular data confirmed that sampled lesions that locally were identified as “bolbo” or “moora”, were effectively CL lesions. Variables were defined prior to the data collection, and their operational definitions were stated in the methods section of the manuscript (scoring).

How the attitude part of the questionnaire was done is unclear. It is mentioned that likert scales were done, ranging from 0 (strongly disagree) to 5 (strongly agree), but that the total attitude score ranged from 0-5. I would assume a maximum score of 25 would be possible if a patient strongly agreed with all statements? The attitude questions are classified as having unfavourable attitude and favourable attitude, but it is unclear what is really meant by this as the term is vague and seems to be more about treatment than the disease itself. Why is considered negative if a disease is not treatable or a health problem or not? I would suggest to simply describe attitudes and not classify them as good and bad.

Response: We appreciate the reviewer’s suggestions/comments. Our analysis is the other way (and widely used) of the reviewer’s suggestion. We created a composite variable with the scales mentioned. A respondent was assigned a maximum score of 5 when he/she strongly agreed with all questions and 0 when strongly disagreed with all questions. We created the composite variable in this way. The scoring methods were presented in the manuscript (page 7, line 156). The composite variables better indicated the cumulative KAP responses. Then we also pointed out our view in that it sounds better if we measure the degree of attitude (as good, bad, favorable, unfavorable, etc) than by giving a simple description. There are previously published reports that used similar methods and analyses (Devipriya et al., 2021 (PLoS One), Ahmad et al., 2022 (PLoS One), and Kebede et al., 2016 (Asian Pacific Journal of Tropical Biomedicine)). We hope that the revisions made to the paper shape it in a way the reviewer can accept.

Similarly, for practices I would advise to simply describe, rather than calling it good or bad practices. Why is it good or bad practice for CL prevention to live with domestic animals? Why is it poor practice to go to traditional healers?

Response: CL is a treatable disease. However, the attitude of the respondents was assessed, and a portion of them perceived it as “not treatable”. When we assessed the attitude towards the use of modern medication (which we consider a “good practice”), the attitude was not in favor of modern medication.

In addition, I think asking about CL treatment and prevention practices is more appropriately asked to CL patients rather than community members.

Response: Thanks again for the opinion. We explained our choice to opt for a community-based approach already mentioned above.

The authors do a regression analysis to look at factors associated with CL knowledge. I would advise them to remove this, as it doesn’t really make sense. Especially since the population is not representative, the classification into good or poor is not strong, and the rationale of it is not clear.

Response: We addressed the population representativeness in our survey in the response above. The regression analysis in relation to the CL knowledge showed significant/interesting associations, particularly with the districts that can be helpful to evidence-based action/intervention. Its importance was also mentioned by the other reviewer, and we were commented to include it in the abstract. So we decided to keep the analysis.

The discussion is a bit long, and there is some repetition. I would advise the authors to make it more concise. Important things to add are a section on limitations and the relevance of this manuscript.

Response: We appreciate the reviewer for the valuable suggestions/comments. We accepted the comment and revised the discussion; we also included the study limitations in the manuscript.

MINOR COMMENTS (also see PDF)

Were there any CL patients among the recruited participants?

Response: Yes, but we did not make any analysis in terms of CL patients, as our study population was the household heads in the general community.

It is not clear what the sample size is based on, is that knowledge? Attitude? Practices? This should be specified

Response: we haven’t separately calculated the sample size for knowledge, attitude, and practices. We calculated the largest sample size that could be obtained with the formula (mentioned above and in the MS).

Something more about the burden of CL in this area should be explained, what is the endemicity of the two districts and the villages that were sampled?

Response: It needs a separate study to report the burden/ endemicity level of the districts and the villages. We investigated that question in a separate paper that will be published later.

Classification of variables is not clearly explained. Why was age cutoff of 40 used? How was no formal education classified? Why was a family size of 5 used as a cutoff?

Response: The sociodemographic variables were classified in order to provide logical comparisons based on the nature of our study population. The age category is because the participants were adults (household heads) who had higher ages. To clarify “formal education”, reference (table footnote) is included under the table in the revised manuscript. The family size cutoff is reasonably classified as the average size of the households in the study area is nearly 5.

**Reviewer #3**

Thank you for the opportunity to review the manuscript titled “Knowledge, attitude, and practice of the rural community about cutaneous leishmaniasis in Wolaita zone, southern Ethiopia”. I would like to appreciate the authors' effort to undertake this valuable task. Based on my point of view I raised the following points that I believed might improve the manuscript. This work plans to assess the community's knowledge, attitude, and practice about cutaneous leishmaniasis in Wolaita zone, southern Ethiopia. This kind of researches provides baseline information for future studies. However, there are some concerns which when addressed hope to improve the quality of the manuscript. Find my specific comments below.

Comment #1- Line 42, “What plants were used in herbal treatment?

Response: We appreciate the reviewer for the question. In this work, we didn’t study the type of plants used to treat the disease. Because, the herbal medicinal plants are known by the traditional healers (individual practitioners) and not by community members. So, in this particular study, we didn’t approach the traditional healers to obtain information about the plants used for treatment.

Comment #2- Line 54, “Cutaneous leishmaniasis…. For the first time, write it completely and then use its abbreviation (CL)

Response: We accepted the comment.

Comment #3- Line 63, “In 2020, about 85% of new….. The reference related to this sentence is for 2010.

Response: We accepted the comment and addressed it in the revised manuscript.

Comment #4- Line 65, East African countries, including Ethiopia, are…… There is a duplicate with the last paragraph of the introduction.

Response: We accepted the comment; we revised the paragraphs.

Comment #5- Table 1: Instead of level of education I would recommend use of “years of education”. Reads might not be familiar with Ethiopian system of education.

Response: We are thankful to the reviewer for the recommendation. However, the data we collected for this variable (education) was not in the “year/s” of education of the respondents. If the reviewer is concerned about the technicality of the word “level”, we used “status” instead in the revised manuscript to contextualize the word.

Comment #6- Did you have any missing data among the variables?

Response: No.

Comment #7- I suggest the authors put raw data in an online open access repository for preserve and share their research outputs.

Response: We accepted the suggestion. We are happy to do that. For the ethical concern, we will remove any personal identifying information from the raw data.

Comment #8- Line 251, “ perhaps??? In the south of Ethiopia, there are similar studies, please write clearly whether there is a similar study in your studied area.

Response: There is no similar study in our study area. We revised the paragraph.

References

9- Italicize the scientific names (Leishmania tropica in REF 13)

10- Journal names should appear in abbreviation.

11- No journal name should start with "the"

12- Books need city.

Response: We appreciate the reviewer for the comments. We addressed the comments in the revised manuscript.
