# Supplementary material for: Knowledge, attitude, and practice of the rural community about cutaneous leishmaniasis in Wolaita zone, southern Ethiopia
Source: PLoS One. 2023 Mar 28;18(3):e0283582. doi: 10.1371/journal.pone.0283582 (PMC10047512; doi:10.1371/journal.pone.0283582)
Supplement: Attachment — Submitted filename: Response to reviewers.docx [file pone.0283582.s006.docx]

Response to Reviewers (PLOS ONE)

Manuscript Number: PONE-D-22-15781R1

**Knowledge, attitude, and practice of the rural community about cutaneous leishmaniasis in Wolaita zone, southern Ethiopia**

We thank the editor and the reviewers for giving us feed-backs on the revisions made so far. We also appreciate the second review through which we received comments/suggestions to further construct our manuscript. Please kindly find below our responses to the points raised in the second review. In this response letter, the editor’s and the reviewer’s comments/suggestions are in blue, and our responses are in black. We have also made the requested changes in the manuscript (yellow colored). We hope the revisions now address the comments the academic editor and the reviewer pointed out.

**Academic Editor's Comments:**

Journal Requirements:

Comment #1. Please review your reference list to ensure that it is complete and correct. If you have cited papers that have been retracted, please include the rationale for doing so in the manuscript text, or remove these references and replace them with relevant current references. Any changes to the reference list should be mentioned in the rebuttal letter that accompanies your revised manuscript. If you need to cite a retracted article, indicate the article’s retracted status in the References list and also include a citation and full reference for the retraction notice.

Response: Thank you for the concern about the completeness and correctness of references. We ensure that all references are complete and correct and all are relevant. There are no retracted papers cited in the manuscript.

**Reviewers' Comments:**

Reviewer #1: All the comments have been addressed by the authors and I recommend the manuscript for publication in PLOS One.

Response: We thank the reviewer for accepting our revisions and recommending the manuscript for publication.

Reviewer #2: Most of the comments have been adequately adressed.
I still have two main points that I am not satisfied with
-I still really disagree with categorizing attitudes as positive or negative. Firstly, I don't what see it adds to classify it overall, and secondly, the overall category (negative/positive attitude towards CL) doesn’t fit the underlying questions.
To me, classifying someone as having a positive or negative attitude is possible (e.g. the last two questions). But the questions about CL being treatable, CL patients having to receive modern medication and CL being a health problem don’t fit the description as good/bad attitude. Why is it negative attitude if someone considers CL a health problem?
How can you classify whether people CL as a health problem as positive or negative? And how can you classify CL is treatable

Response: We thank the reviewer for the comments. We have now addressed the comments of the reviewer by descriptively stating the attitude section (line 202 to 207 of the revised manuscript).

-The discussion is still very long and contains sections which are more suited for results.
Try to really link it to things that can be used for policy and practice and how the findings can be use to improve community engagement.

Response: We accepted the comments and made revisions to the manuscript now.

I have made suggestions in the attached PDF.

Response: Thank you for providing suggestions in the PDF attachment. We revised our paper accordingly (most are accepted /accepted with modifications) for suggestions given in the PDF. However, some explanations/statements considered important for our manuscript are retained as they were in the previous revision.

1) Important statements of rationale in the background section and explanations in the discussion part that suit our findings are maintained as in the previous revision.

For example, a) explanation for the study's rationale in the last paragraph of the introduction. CL/KAP study in Ochollo vs Wolaita Zone (current study area): different communities may have different perceptions, practices and knowledge about the diseases. the Ochollo and Wolaita Zone are two different places where the socio-economy and the socio-culture are different. There is also a difference in particular to CL prevalence and its impact. Unlike our study area, Ochollo is subjected to several CL-related studies and community education that exposed the Ochollo community to better CL awareness. b) in line 249 to 255 of the revised manuscript, the lack of knowledge on CL transmission was specifically addressed in this paragraph. The explanations provided here are for the results of Table 2, and c) in line 284 to 295 of the revised manuscript, the paragraph explains the findings regarding the association of socio-demographic factors to the general CL knowledge.

2) Reviewer’s suggestions for limitations: A) We believe that assessing the KAP of CL patients is another researchable idea/perspective (with its distinct objectives) than considering it as a limitation of the current study. We have indeed justified (in the previous response letter) the appropriateness of household heads for the KAP study by providing published articles (Tesfaye *et al*., 2021 (BMC Tropical Diseases, Travel Medicine and Vaccines); Sarkari *et al.,* 2014 (Asian Pacific Journal of Tropical Biomedicine); Alemu *et al.,* 2013 (BMC Public Health). Studying the KAP of CL patients was not our objective. B) The endemicity and prevalence of CL in the area are under study. As we said in the previous response letter, we are assessing these in our undergoing research that will later be finalized and published in a separate paper.
