# Supplementary material for: Knowledge, attitude, and practice of the rural community about cutaneous leishmaniasis in Wolaita zone, southern Ethiopia
Source: PLoS One. 2023 Mar 28;18(3):e0283582. doi: 10.1371/journal.pone.0283582 (PMC10047512; doi:10.1371/journal.pone.0283582)
Supplement: Attachment — Submitted filename: Response to reviewers.docx [file pone.0283582.s007.docx]

Response to Reviewers (PLOS ONE)

Manuscript Number: PONE-D-22-15781R2

**Knowledge, attitude, and practice of the rural community about cutaneous leishmaniasis in Wolaita zone, southern Ethiopia**

We thank the editor and the reviewers for giving us feed-backs on the manuscript. We also appreciate PLoS One/editor for allowing us to clean our manuscript further. Please find below our responses to the points raised in the current review. In this response letter, the editor’s and the reviewer’s comments/suggestions are in blue, and our responses are in black. We have also made the requested changes in the manuscript (yellow colored). We hope the recent revisions now address the comments the academic editor and reviewer pointed out.

**Academic Editor's Comments:**

Journal Requirements:

Comment #1. Please review your reference list to ensure that it is complete and correct. If you have cited papers that have been retracted, please include the rationale for doing so in the manuscript text, or remove these references and replace them with relevant current references. Any changes to the reference list should be mentioned in the rebuttal letter that accompanies your revised manuscript. If you need to cite a retracted article, indicate the article’s retracted status in the References list and also include a citation and full reference for the retraction notice.

Response: Thank you for the concern about the completeness and correctness of references. We ensure that all references are complete and correct and all are relevant.

**Reviewer’s Comments:**

Reviewer #2: Thank you for your revised manuscript. I have no major comments left. Here I provide a few remaining suggestions to improve readability:

• Line 205-207: Here for binary responses both percentages are given (the n,% for those who had bad feelings and n,% for those who didn’t). This can be reduced as follows.
A significant number (185,205 43.8%) of the respondents had bad feelings about meeting CL patients. Most (70.9 %) respondents believed CL was not a stigmatizing disease (Table 3).

Response: We thank the reviewer for the comments and suggestions. We have accepted the comments/suggestions (lines 206 to 208 of the revised manuscript).

• Line 260: only one third thought CL to be stigmatizing. This seems low. It would be interesting to explain potential reasons for this, rather than stating things that can lead to stigma.

Response: We appreciate the reviewer for this suggestion. We revised the section (lines 259 to 263).

• Line 273: there seems to be a typo, should be Kindo Didaye (not Kndo)

Response: Thank you. The typo is corrected (line 273).

• Line 289: "Studies also reported different findings concerning CL knowledge and age-wise association [18, 29]." It would be better to specify if the findings confirm your finding or contrast it.

Response: We accepted the comment. Comparisons to the findings are specified (lines 289 to 293).

o Ref 18 shows a relationship with age, but doesn’t show a difference in those below and above 40

Response: We revised it now (lines 289/290).

o Ref 29 doesn’t show a relationship with age.

Response: Ref 29 presents the CL knowledge and age-wise association (on page 9, Table 6 of the reference paper). In the reference paper, age >40 was associated with CL knowledge, whch contrasts our finding (lines 291/292).

o Suggestion to rewrite: A relationship between CL knowledge and age was also shown in Tigray [18], although different age categories were used.

Response: Thanks. We accepted the suggestion (lines 289/290).

• Line 300: better to replace misinterpreted with 'generalized'

Response: We accepted the suggestion (line 303).

• Line 311: campaign(s), should be plural

Response: We accepted the comment (line 313).

• Some references should be checked
o Ref 5 WHOECotCot?
o Ref 15: initials should come after the last name
o Ref 28: WHO is not an author here

Response: Thanks. References are checked. We revised the section.
